# Accumulation of V_H_ Replacement Products in IgH Genes Derived from Autoimmune Diseases and Anti-Viral Responses in Human

**DOI:** 10.3389/fimmu.2014.00345

**Published:** 2014-07-22

**Authors:** Miles D. Lange, Lin Huang, Yangsheng Yu, Song Li, Hongyan Liao, Michael Zemlin, Kaihong Su, Zhixin Zhang

**Affiliations:** ^1^Department of Pathology and Microbiology, University of Nebraska Medical Center, Omaha, NE, USA; ^2^Department of Pediatrics, Philipps-University Marburg, Marburg, Germany; ^3^The Eppley Cancer Institute, University of Nebraska Medical Center, Omaha, NE, USA; ^4^Department of Internal Medicine, University of Nebraska Medical Center, Omaha, NE, USA

**Keywords:** B-cell, antibody, IgH genes, cryptic RSS, V_H_ replacement, V_H_ replacement footprint, autoimmune disease, anti-viral response

## Abstract

V_H_ replacement refers to RAG-mediated secondary recombination of the IgH genes, which renews almost the entire V_H_ gene coding region but retains a short stretch of nucleotides as a V_H_ replacement footprint at the newly generated V_H_–D_H_ junction. To explore the biological significance of V_H_ replacement to the antibody repertoire, we developed a Java-based V_H_ replacement footprint analyzer program and analyzed the distribution of V_H_ replacement products in 61,851 human IgH gene sequences downloaded from the NCBI database. The initial assignment of the V_H_, D_H_, and J_H_ gene segments provided a comprehensive view of the human IgH repertoire. To our interest, the overall frequency of V_H_ replacement products is 12.1%; the frequencies of V_H_ replacement products in IgH genes using different V_H_ germline genes vary significantly. Importantly, the frequencies of V_H_ replacement products are significantly elevated in IgH genes derived from different autoimmune diseases, including rheumatoid arthritis, systemic lupus erythematosus, and allergic rhinitis, and in IgH genes encoding various autoantibodies or anti-viral antibodies. The identified V_H_ replacement footprints preferentially encoded charged amino acids to elongate IgH CDR3 regions, which may contribute to their autoreactivities or anti-viral functions. Analyses of the mutation status of the identified V_H_ replacement products suggested that they had been actively involved in immune responses. These results provide a global view of the distribution of V_H_ replacement products in human IgH genes, especially in IgH genes derived from autoimmune diseases and anti-viral immune responses.

## Introduction

To protect our body from various infectious agents, the adaptive immune system has evolved the capability to generate a vast number of antibody (Ab) specificities through somatic rearrangement of previously separated variable (V), diversity (D) (for heavy chain only), and joining (J) gene segments to form the variable domain exons of immunoglobulin genes (1–3). V(D)J recombination is catalyzed by a pair of recombination activating gene products (RAG1 and RAG2) (4–6). Specific joining of the V, D, and J gene segments is directed by the recombination signal sequences (RSS) flanking each rearranging gene segment (7). The RSS is composed of a highly conserved heptamer (5’-CACTGTG-3’) and a nonamer (5’-ACAAAAACC-3’) separated by a non-conserved spacer region with either 12 or 23 bp in length (7–9). There are 44 functional V_H_ genes, 27 D_H_ genes, and 6 J_H_ genes within the human IgH locus. The diversified IgH repertoire is generated at different levels, including the random recombination of V, D, and J genes segments, imprecise processing of the coding-ends, addition of non-template nucleotides by terminal deoxynucleotidyl transferase (TdT), random pairing of IgH with Igκ or Igλ light chains, and later through somatic hypermutation and class switch recombination during antigen dependent germinal center reaction (2). Previous analyses of the IgH repertoire have provided important information regarding the developmental process and function of B lineage cells (10, 11). For examples, earlier studies on the expression and rearrangement status of IgH genes demonstrated that IgH gene are rearranged sequentially during early B lineage cell development, in which D_H_ to J_H_ rearrangements occurs prior to V_H_ to DJ_H_ rearrangements followed by rearrangement of the Igκ and then Igλ light chain genes (12, 13). Analyses of the Ig gene repertoires of different autoimmune diseases such as rheumatoid arthritis (RA) and systemic lupus erythematosus (SLE) revealed skewed usages of specific germline V_H_ genes (14–16), unusually long CDR3 regions within the IgH and IgL genes (17, 18), and accumulation of somatic hypermutation in the variable regions of IgH and IgL genes (15, 19).

The random process of V(D)J recombination is essential for generating a diverse IgH repertoire, however, it also produces non-functional IgH genes or IgH genes encoding autoreactive antigen receptors (2, 20). Early B lineage cells carrying non-functional IgH rearrangements must re-initiate the V(D)J recombination process to generate functional B-cell receptors (BCRs) for subsequent development; on the other hand, B-cells expressing autoreactive receptors will be removed from the repertoire through receptor editing, clonal deletion, or anergy to establish central tolerance (1, 21, 22). Receptor editing refers to RAG-mediated secondary recombination of previously rearranged IgH or IgL genes (1, 21, 22). The organizations of the Igκ and Igλ loci allow continuous secondary recombination by joining an upstream V_L_ gene with a downstream J_L_ gene segment. The previously formed V_L_J_L_ joints are deleted during secondary recombination leaving no trace in the newly formed V_L_J_L_ junctions; the only indication of extensive light chain gene editing is the elevated usage of the 3′ Jκ or Jλ genes and the deletion of the Igκ locus (23, 24).

The unwanted IgH genes can also be changed through a RAG-mediated V_H_ replacement process using the cryptic recombination signal sequences (cRSSs) embedded within the framework-3 regions of previously rearranged V_H_ genes (21, 22, 25). The concept of V_H_ replacement was originally proposed to explain the observation that functional IgH genes were generated in mouse pre-B-cell leukemia lines initially harboring non-functional IgH rearrangements (26–28). Comparison of the functional IgH genes versus the non-functional IgH rearrangements suggested a V_H_ to V_H_DJ_H_ recombination process mediated by the cRSS sites (26, 27). Subsequently, the occurrence of V_H_ replacement had been demonstrated in mouse models carrying knocked-in IgH genes encoding anti-DNA Abs, anti-NP Abs, or non-functional IgH genes in both alleles (29–34). Despite these findings, the natural occurrence of V_H_ replacement during early B-cell development in mouse remains to be determined (35, 36).

Ongoing V_H_ replacement in human B-cells had been found in a human leukemia cell line, EU12, by detection of RAG-mediated cRSS double stranded DNA breaks (DSBs) and by amplification of different V_H_ replacement excision circles (37). The detection of DSBs at the V_H3_–cRSS borders in human bone marrow immature B-cells provided the first evidence for the natural occurrence of V_H_ replacement during normal B-cell development in humans (37). The occurrence of V_H_ replacement in bone marrow immature B-cells is consistent with the observation that RAG1 and RAG2 genes can be reinduced in these cells to catalyze IgL gene editing (24, 38, 39). Our recent studies showed that V_H_ replacement occurs in the newly immigrated immature B-cells in the peripheral blood of healthy donors, which can be further induced through BCR-mediated signaling in Ref. (40). The cRSS-mediated V_H_ replacement was of particular interest because the cRSS motifs are found in 40 out of 44 human V_H_ germline genes and in the majority of mouse V_H_ germline genes (22, 41). V_H_ replacement renews almost the entire V_H_ gene coding region but retains a short stretch of nucleotides as a V_H_ replacement footprint at the V_H_–D_H_ junction (37). Such footprints can be used to identify V_H_ replacement products through analysis of IgH gene sequences. The initial analyses of 417 human IgH gene sequences estimated that V_H_ replacement products contribute to about 5% of the normal IgH repertoire (37). Interestingly, analyses of the amino acids encoded by the V_H_ replacement footprints revealed that these footprints preferentially contribute charged amino acids into the IgH CDR3 regions, which is different from the low frequency of charged amino acids encoded by human germline D_H_ genes or N region sequences added by TdT (37).

To explore the biological significance of V_H_ replacement, we developed a Java-based computer program and analyzed 61,851 human IgH gene sequences from the NCBI database to determine the distribution of V_H_ replacement products.

## Materials and Methods

### Development of the V_H_ replacement footprint analyzer program

The V_H_ replacement footprint analyzer (V_H_RFA) program was developed using the NetBeans 7.01 IDE with Java development kit (JDK) and tested under Windows, Mac OS X, and Ubuntu Linux (42). The reference human V_H_ germline gene sequences were downloaded from the IMGT database to generate the library of V_H_ replacement footprints with different lengths. For the initial test of the V_H_RFA program, we used 417 IgH sequences that had been analyzed in our previous study to manually identify potential V_H_ replacement products (37, 43). The 61,851 human IgH gene sequences were downloaded from the NCBI database on April 20, 2011.

### Analysis of IgH gene sequences and identification of potential V_H_ replacement products using the V_H_RFA program

The IgH gene sequence files from NCBI database were first converted into FASTA files and uploaded to the V_H_RFA program. The V_H_, D_H_, and J_H_ germline gene usages were assigned by automatic submission of sequences in batches to the IMGT/V-Quest program (http://www.imgt.org/IMGT_vquest/share/textes/) (44) and the results were exported as Microsoft Excel files to a local computer. Identical IgH gene sequences in the original NCBI database were removed based on their V_H_–D_H_–J_H_ junctions and the remaining 39,438 unique human IgH gene sequences with identifiable V_H_, D_H_, and J_H_ genes were further analyzed to identify potential V_H_ replacement products and calculate the frequencies of V_H_ replacement products in subsequent analyses. Briefly, the IgH gene sequences with clear identifiable V_H_, D_H_, and J_H_ genes were analyzed to identify V_H_ replacement footprints with 7, 6, 5, 4, and 3-mer V_H_ replacement footprint motifs at their V_H_–D_H_ junction (N1) regions and D_H_–J_H_ junction (N2) regions. The frequency of V_H_ replacement products was calculated by dividing the number of IgH genes with V_H_ replacement footprints in the N1 regions with the total number of unique IgH gene sequences. IgH genes with 7, 6, 4, and 3-mer V_H_ replacement footprint motifs within their N1 regions were also analyzed and discussed. The positive prediction value with 95% confidence interval using the 6, 5, 4, and 3-mer V_H_ replacement footprint motifs to assign V_H_ replacement products are 68, 59, 54, and 52%, respectively. In the following comparison, the V_H_ replacement products mainly refer to IgH genes with 5-mer V_H_ replacement footprint within their N1 regions.

The distribution of V_H_ replacement products in IgH genes derived from different keyword sub-categories were analyzed based on the information linked to each sequence in the NCBI GenBank files. The frequencies of V_H_ replacement products with pentameric footprints were used for all these comparisons. For mutational analysis the IgH gene sequences had a minimum of ≥80% nucleotide similarity to the assigned germline V_H_ gene sequences.

### Statistical analysis

Statistical significance was determined by using either the two-tailed Chi square test with Yates’ correction or the unpaired *t*-test. *p* < 0.05 is considered statistically significant and *p* < 0.0001 is considered extremely statistically significant.

## Results

### Differential usage of germline V_H_, D_H_, and J_H_ genes in human IgH gene sequences

**Figure 1:**
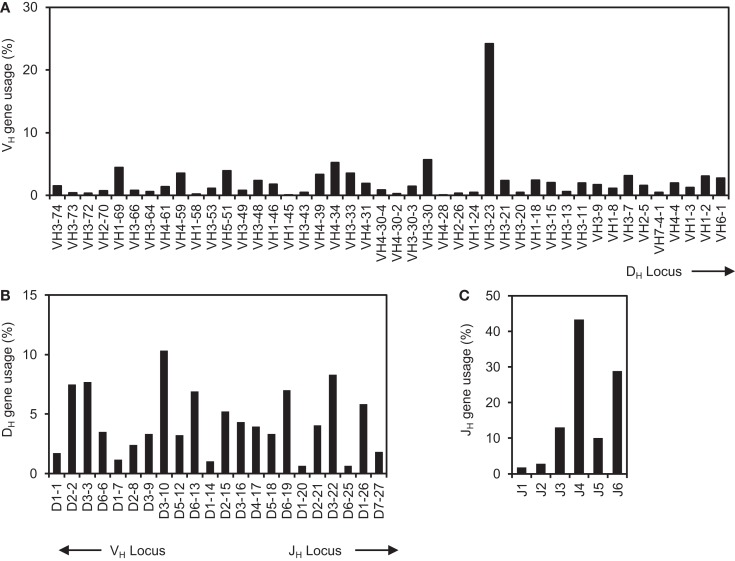
**The comprehensive analysis of human IgH repertoire**. The 61,851 human IgH gene sequences were downloaded from the NCBI database on May, 2012. The sequences were first analyzed for their V_H_, D_H_, and J_H_ gene usage using the IMGT/V-Quest program and the identical sequences were removed. The frequencies of V_H_ **(A)**, D_H_ **(B)**, and J_H_ **(C)** germline gene usages in the 39,438 unique human IgH gene sequences were shown.

We have developed a Java-based V_H_RFA computer program to analyze large number of IgH gene sequences and to identify potential V_H_ replacement products (42). In the current study, the 61,851 human IgH gene sequences were downloaded from the NCBI database. The initial analysis showed that 54,970 IgH genes have identifiable V_H_, D_H_, and J_H_ gene segments. After removal of duplicate IgH sequences, the remaining 39,438 unique IgH genes with identifiable V_H_, J_H_, and D_H_ genes were further analyzed. The usages of the V_H_, J_H_, and D_H_ germline genes in these sequences represent a combinatorial view of the human IgH repertoire from many studies (Figure 1). The usages of all the 44 functional human germline V_H_ genes were confirmed in this dataset (Figure 1A); the frequencies of individual V_H_ germline gene usage varied considerably. For different families of V_H_ genes, the V_H_3 family of genes was predominantly utilized, followed by the V_H_4 and V_H_1 families of genes (Figure 1A). Such results are consistent with previous analyses of small groups of IgH gene sequences, Among individual V_H_ genes, the V_H3–23_ gene was used the most frequently in 9536 IgH genes (25%). The V_H4–28_ gene was used less frequently, which was only found in 13 IgH rearrangements (0.03%). The differential usages of individual V_H_ germline genes did not seem to correlate with their relative location within the IgH locus (Figure 1A). Within the IgH locus, the V_H1–24_, V_H2–26_, and V_H3–30_ genes are located very close to the V_H3–23_ and V_H4–28_ genes. However, the frequency of the V_H3–23_ gene usage is only 4-fold higher than those of the V_H3–30_ gene, but is 50- and 80-fold higher than that of the V_H1–24_ and V_H2–26_ genes, respectively (Figure 1A).

Among different D_H_ genes, the D_H3_ gene family was predominantly used in 35% of IgH genes, in which the D_H3–10_, D_H3–3_, and D_H3–22_ genes were used frequently; The D_H1_ gene family was used less frequently (Figure 1B). Among J_H_ germline genes, the J_H4_ gene was predominantly used followed by the J_H6_ gene (Figure 1C). These results are consistent with previous individual reports with small number of IgH sequences. Taken together, this analysis provides a comprehensive view of the existing human IgH gene sequences in the NCBI database.

### Identification of V_H_ replacement products using the V_H_RFA program

To identify potential V_H_ replacement products in a large number of IgH gene sequences, the V_H_RFA program first generated libraries of potential V_H_ replacement footprint database with different length based on the V_H_ gene 3′ ending sequences following the conserved cRSS sites of all the functional human V_H_ germline genes (Tables S1 and S2 in Supplementary Material). Then, the V_H_RFA program uses these libraries to search for the presence of V_H_ replacement footprint motifs with specified lengths at the V_H_–D_H_ junction (N1) regions or the D_H_–J_H_ junction (N2) regions of IgH genes. As an initial test of the newly developed V_H_RFA program, we reanalyzed the 417 human IgH gene sequences that had been to manually identify potential V_H_ replacement products analyzed in a previous study (37). The V_H_RFA program efficiently identified V_H_ replacement footprint motifs with 3, 4, 5, 6, or 7 nucleotides in both the N1 and N2 regions (Table 1, top). The frequencies of V_H_ replacement footprint motifs with 3, 4, or 5-mer in the N1 regions are significantly higher than those in the N2 regions (Table 1, top), indicating that the addition of such motifs in the N1 region is not a random event. Based on the identification of 5-mer V_H_ replacement footprints, 7.3% of the IgH gene sequences can be assigned as potential V_H_ replacement products. Further review of these IgH genes confirmed the identified pentameric V_H_ replacement motifs within the V_H_–D_H_ junctions (Table 2, N1 regions). If we consider the 4- or 3-mer V_H_ replacement footprints within the N1 regions, 25 or 54.7% of IgH genes can be assigned as potential V_H_ replacement products, respectively (Table 1; Table S3 in Supplementary Material). These results are consistent with our previously manual assignment of V_H_ replacement products in this group of IgH genes and provide the first validation of the V_H_RFA program.

**Table 1:** **Frequencies of V_H_ replacement footprint motifs in the N1 and N2 regions of human IgH genes**.

	Total number of sequences	Sequences with V_H_, D_H_, J_H_ gene assignment^a^	Length of V_H_ replacement footprint	V_H_ replacement footprint motifs in N1	V_H_ replacement footprint motifs in N2	*p*-Value^b^	Frequency of V_H_ replacement products (%)^c^
Test IgH sequences^d^	417	396	3	217	140	0.0001	54.7
			4	99	64	0.0028	25.0
			5	29	15	0.0437	7.3
			6	5	3^e^	NA^f^	NA^f^
			7	2	0	NA^f^	NA^f^
NCBI IgH sequences^g^	61,851	39,438	3	23,195	20,699	0.0001	58.8
			4	13,365	11,240	0.0001	33.9
			5	4788	3499	0.0001	12.1
			6	1490	813	0.0001	4.3
			7	382	140	0.0001	1.1

*^a^Unique IgH gene sequences with identifiable V_H_, D_H_, and J_H_ genes were analyzed. These IgH sequences contain both functional and non-functional IgH rearrangements. N1, V_H_–D_H_ junction regions; N2, D_H_–J_H_ junction regions*.

*^b^The frequencies of V_H_ replacement footprint motifs with different length within the N1 or the N2 regions were compared by two-tailed Chi square with Yates’ correction. *p* < 0.05 is considered statistically significant and *p* < 0.0001 is considered extremely statistically significant*.

*^c^The frequency of V_H_ replacement products was calculated using the number of sequences with V_H_ replacement motifs with different length in the N1 regions divided by the total number of unique IgH gene sequences*.

*^d^These IgH gene sequences had been analyzed manually for V_H_ replacement products (37)*.

*^e^All the three 6-mer footprints within the N2 regions could be due to second D_H_ gene segments*.

*^f^Not applicable*.

*^g^The human IgH gene sequence dataset was downloaded from the NCBI database on April 20, 2011*.

**Table 2:** **Identification of potential V_H_ replacement products in human IgH sequences**.

Accession No.	V_H_ gene	V_H_	P	N1^a^	P	D_H_	CDR3 (aa)^b^
AF235818	VH1-69*06	tgtgcgaga		gaagcaaagtttgagaag		gctgccaaacc	AREAKFEKAAKPYYYYGMDV
AF235903	VH3-33*01	tgtgcgagaga		cagac		agctgctgctgg	ARDRQLLLGYGMDV
AF235823	VH3-11*01	tgtgcgagaga		caccctcacgaaatcacc		ttacgatttttggagtggttattat	ARDTLTKSPYDFWSGYYGLTYYYYGMDV
AF235857	VH3-23*01	tgtgcgaaaga	t	gaagaggag		tattgtggtagaaccagctgct	AKDEEEYCGRTSCFCMDV
AF235601	VH1-18*01	tgtgcgagaga		cgacggacgggcggcgg		attgtagtggtggtagctgctactcc	ARDDGRAADCSGGSCYSDY
AF235609	VH3-33*05	tgtgcgaga		agagggccaatcc		atatcagcagctgg	ARRGPIHISSWYYYYYGMDV
AF235766	VH3-30*03	tgtgcga		aacagtggacgc		atattgtgg	AKQWTHIVVFDI
AF235806	VH3-15*01	tgt		cattcggggggtagacc		gtatagcagtggctggt	HSGGRPYSSGWSPKWYYGMDV
AF235787	VH3-23*01	tgtgcgaaaga	tc	aacctcgaaag		gcagcagctggta	AKDQPRKAAAGMYYYGMDV
AF235574	VH4-59*07	tgtgcgaga		cgaaat		tattactatgatagtagtggt	ARRNYYYDSSGPDAFDI
AF235726	VH1-69*06	tgtgcg		gggagaggagagtat		ggctatagcagcagctgg	AGRGEYGYSSSWFDY
AF235869	VH2-70*10	tgtgc		cagaca		atattgtggtggtgactgct	ARQYCGGDCCSDY
AF235809	VH4-39*07	tgtgcga		caaaatc	c	gtattacgatattttgactggttatt	ATKSVLRYFDWLLPSYYYYYGMDV
AF235610	VH3-30-3*01	tgtgcgaga		gatgaaag		tagcagtggctgg	ARDESSSGWYWYFDL
AF235541	VH3-48*03	tgtgcgagaga	tc	gacgcgaccggat		taactgggga	ARDRRDRINWGYYYGMDV
AF235758	VH2-70*01	tgtgcacggata		agggccctagacgta		aactgggga	ARIRALDVNWGGWYFDL
AF235544	VH3-66*01	tgtgcgagaga	tc	gagac		tacgatttttggagtggtt	ARDRDYDFWSGYAFDI
AF235692	VH3-33*01	tgtgcgagaga		gggggagattgat		catattgtggtggtgactgctatccc	AREGEIDHIVVVTAIPNWFDP
AF235764	VH1-3*01	tgtgcgagag		cgaga	ct	aggatattgtagtggtggtagctgctactcc	ARARLGYCSGGSCYSGGFDY
AF235793	VH1-69*02	tgtgcgaga		gatctcacttacgggc		attttgactggtta	ARDLTYGHFDWLPPHYYYYYGMDV
AF235897	VH3-21*01	tgtgcgaga		tcaacggcatca		tacggtgactac	ARSTASYGDYDNWFDP
AF235796	VH3-30*03	tgtgcgaaaga	tc	ctacgggaaccacaaacttatctcccttagggcg		agcagcagct	AKDPTGTTNLSPLGRAAAYVYYYYYGMDV
AF235588	VH4-59*08	tgtgcga		cccatcggat		taactgggga	ATHRINWGFDY
AF235907	VH5-51*01	tgtg		tgcgagacagctcg		tacagctatggtt	VRDSSYSYGLSNLYYYGMDV
AF235842	VH3-23*01	tgtgcgaaaga	t	ttcccagacgagcccgg		gtaccagctgctatac	AKDFPDEPGYQLLYGSLDY
AF235812	VH5-a*01	tgtgcgag		ggccgaaatcttatccgg		agcagtggc	ARAEILSGAVAPRDY
AF235657	VH5-51*01	tgtgcgagac		gagaacaacc		tgggacccact	ARREQPGTHLNY
AF235626	VH3-21*01	tgtggga		aagaggacc		ggagttatta	GKEDRSYYDY
AF235565	VH3-23*01	tgt		accacagacccggccttgaggacctc		actgctggggt	TTDPALRTSLLGSFDY

*^a^ The identified V_H_ replacement footprints are underlined and highlighted in *red* in the N1 regions*.

*^b^ The amino acids encoded by the identified V_H_ replacement footprints are underlined in the amino acid sequences of the CDR3 regions*.

### Contribution of V_H_ replacement products to the human IgH repertoire

With the help of the V_H_RFA program, we searched for potential V_H_ replacement products in the 39,438 unique human IgH sequences with identifiable V_H_, D_H_, and J_H_ genes from the NCBI database. We first compared the frequencies of V_H_ replacement footprint motifs with 3, 4, 5, 6, or 7 nucleotides within the N1 and N2 regions (Table 1, bottom). The frequencies of 3, 4, 5, 6, and 7-mer V_H_ replacement footprint motifs in the N1 regions are extremely statistically significantly higher than those in the N2 regions (Table 1, bottom, *p* < 0.0001), indicating that the presence of such motifs at the N1 region is likely contributed by V_H_ replacement rather than random nucleotide addition. Among these IgH gene sequences, 12.1% of them contain the 5-mer V_H_ replacement footprint motifs and can be assigned as potential V_H_ replacement products (Table 1, bottom). This number indicates a significant contribution of V_H_ replacement products to the diversification of the human IgH repertoire. If we consider the 4- and 3-mer V_H_ replacement footprints motifs, 33.9 and 55.8% of IgH genes can be assigned as potential V_H_ replacement products (Table 1, bottom).

Within the large number of IgH genes, there are 3818 non-functional IgH gene sequences and 687 of them contain the 5-mer V_H_ replacement footprint motifs in their N1 regions, which can be assigned as potential V_H_ replacement products. The frequency of V_H_ replacement products in non-functional IgH genes (18%) is extremely statistically significantly higher than that in the overall functional IgH genes (*p* < 0.0001, two-tailed Chi square test with Yates’ correction). Identification of V_H_ replacement products in non-functional IgH genes fulfills the prediction that V_H_ replacement is a random process that can generate both functional and non-functional IgH rearrangement products. Taken together, these results uncovered a previously unrealized contribution of V_H_ replacement products to the diversification of human IgH repertoire.

### Distribution of V_H_ replacement products in IgH genes using different V_H_ genes

**Figure 2:**
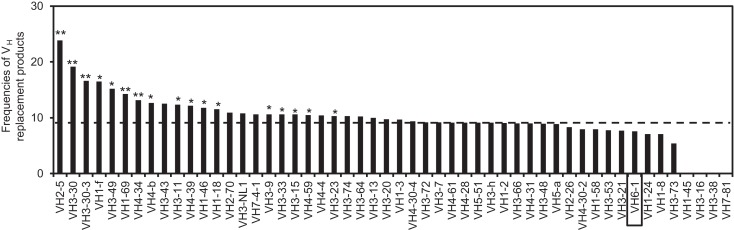
**Distribution of V_H_ replacement products in IgH genes using different V_H_ genes**. The frequencies of V_H_ replacement products in functional IgH genes using each V_H_ germline genes are compared with that in IgH genes using the V_H6-1_ gene. **p < 0.0001, *p < 0.05. The result for IgH genes using the V_H6-1_ gene is highlighted in the box and the frequency of V_H_ replacement products in all the IgH genes is indicated by the dashed line.

Using the V_H_RFA program, we further analyzed the distribution of V_H_ replacement products in IgH genes using different V_H_ genes. The frequencies of V_H_ replacement products in IgH genes using different V_H_ germline genes are different (Figure 2). For examples, the frequencies of V_H_ replacement products in IgH genes using the V_H2–5_, V_H3–30_, V_H3-30-3_, V_H1–69_, and V_H3–34_ genes are 23.88, 19.12, 16.64, 14.28, and 13.13%, which are extremely statistically significantly higher than that in IgH genes using the V_H6-1_ gene (*p* < 0.0001, two-tailed Fisher’s exact test) (Figure 2). As an internal control, 7.56% of IgH genes using the V_H6-1_ gene have 5-mer V_H_ replacement footprints within their N1 regions, which is statistically significantly lower than that in the overall IgH gene sequences (*p* = 0.0004, two-tailed Fisher’s exact test).

### V_H_ replacement products are highly enriched in IgH genes derived from patients with autoimmune diseases or viral infections

**Figure 3:**
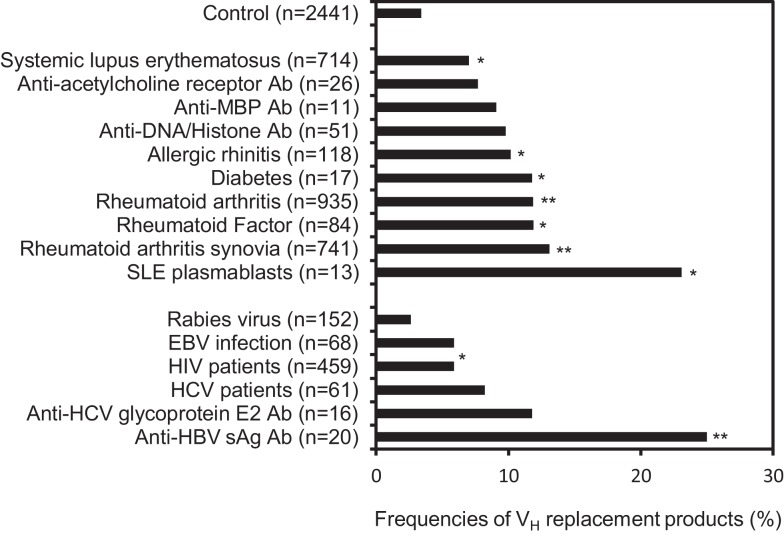
**V_H_ replacement products are significantly enriched in IgH genes derived from autoimmune diseases or viral infections and in IgH genes encoding autoreactive or anti-viral Abs**. Frequencies of V_H_ replacement products in IgH gene sequences derived from different sub-categories were analyzed based on the identification of pentameric V_H_ replacement footprints within their V–D junctions. The frequencies of V_H_ replacement products in IgH genes derived from different autoimmune diseases and viral infections, or in IgH genes encoding auto Abs, anti-viral Abs, or anti-bacterial Abs were compared with that from healthy controls. The number of analyzed IgH gene sequences in each subcategory are indicated (*n*). The arrow head indicates the overall frequency of V_H_ replacement products (12.1%) in the 39,438 unique human IgH sequences. Statistical significance was determined using a two-tailed Chi square test with Yate’s correction. *p < 0.05 is considered statistically significant and **p < 0.0001 is considered extremely statistically significant.

The overall frequency of V_H_ replacement products in the 39,438 unique IgH genes from the NCBI database (12.1%) is much higher than what was estimated in the 417 IgH genes obtained from healthy donors. We reasoned that the majority of IgH gene sequences deposited at the NCBI database was derived from diseased subjects, which may have higher frequencies of V_H_ replacement products. Next, we investigated the distribution of V_H_ replacement products in IgH genes derived from different disease sub-categories. Using the keyword analysis function within the V_H_RFA program, we can correlate the frequencies of V_H_ replacement products with different sub-categories of IgH gene sequences from the NCBI database. For examples, the frequency of V_H_ replacement products in 558 IgH genes derived from healthy donors is 8.6% (Figure 3), which is similar to the result obtained from previous analysis of the 417 IgH gene sequences from healthy donors. Interestingly, the frequencies of V_H_ replacement products in IgH genes derived from subjects with different autoimmune diseases, such as allergic rhinitis, RA, and SLE are statistically significantly higher than that in the healthy donors (Figure 3, *p* < 0.05, two-tailed Chi square test with Yates’ correction; Table S4 in Supplementary Material). The frequencies of V_H_ replacement products are further enriched in IgH genes derived from RA synovium and in IgH genes encoding rheumatoid factors, suggesting that B-cells expressing V_H_ replacement products are positively selected in the RA synovium to encode rheumatoid factors (Figure 3, *p* < 0.05, two-tailed Chi square test with Yates’ correction; Table S4 in Supplementary Material). Similarly, V_H_ replacement products are highly enriched in IgH genes derived from SLE plasmablasts (Figure 3, *p* < 0.05, two-tailed Chi square test with Yates’ correction; Table S4 in Supplementary Material), suggesting that these enriched V_H_ replacement products contribute to the production of autoAbs in SLE.

The accumulation of V_H_ replacement in IgH genes derived from patients with different autoimmune diseases suggested that V_H_ replacement products may contribute to the production of autoAbs. Indeed, further analyses showed that V_H_ replacement products are statistically significantly enriched in IgH genes encoding rheumatoid factors, anti-Rh (D) Abs, and anti-acetylcholine receptor Abs (Figure 3, *p* < 0.05, two-tailed Chi square test with Yates’ correction; Table S4 in Supplementary Material).

To our surprise, the frequencies of V_H_ replacement products are significantly elevated in IgH genes derived from different viral infections. For examples, the frequencies of V_H_ replacement products in IgH genes derived from HIV and HCV infected patients are statistically significantly higher than that in healthy donors (Figure 3, *p* < 0.05, two-tailed Chi square test with Yates’ correction; Table S4 in Supplementary Material). Further analyses showed that the V_H_ replacement products contribute to about 30% of IgH genes encoding anti-HCV glycoprotein E2 Abs or anti-HBVsAg Abs. Such frequencies are statistically significantly higher than that in healthy donors (Figure 3, *p* < 0.05, two-tailed Chi square test with Yates’ correction). Taken together, these results showed that V_H_ replacement products are highly enriched in IgH genes derived from patients with different autoimmune diseases and viral infections.

### V_H_ replacement elongates the IgH CDR3

**Figure 4:**
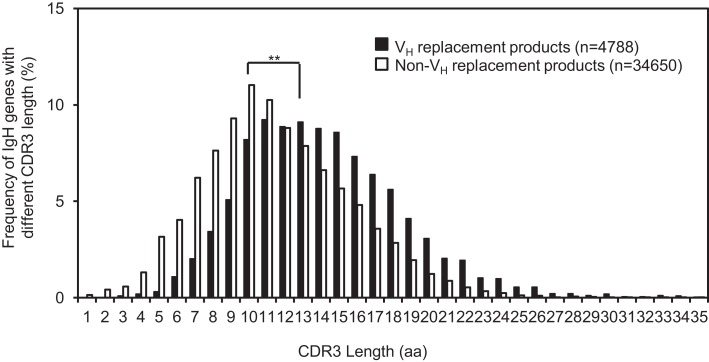
**The average CDR3 length of identified V_H_ replacement products is significantly longer than that of non-V_H_ replacement products**. The distribution of IgH genes with different CDR3 lengths is shown in the bar graph. The average CDR3 length of V_H_ replacement products (black bars) was compared to that of non-V_H_ replacement products (white bars). Statistical significance was determined by using an unpaired *t*-test. **p < 0.0001 is considered extremely statistically significant.

V_H_ replacement renews almost the entire V_H_ coding region. Due to the location of the cRSS site, a short stretch of nucleotides is remained as a V_H_ replacement footprint at the newly formed V_H_–D_H_ junction after the V_H_ replacement process (37). Such V_H_ replacement footprints can contribute up to two amino acids into the IgH CDR3 to elongate the CDR3. The average CDR3 length of the identified V_H_ replacement products is 18.2 ± 5.0 aa (*n* = 4417), which is extremely statistically significantly longer than that of the non-V_H_ replacement products (15.4 ± 4.4 aa, Figure 3, *p* < 0.0001, unpaired *t*-test) (Figure 4). This result confirmed that V_H_ replacement elongates the IgH CDR3 region.

### The V_H_ replacement footprints preferentially encode charged amino acids

**Figure 5:**
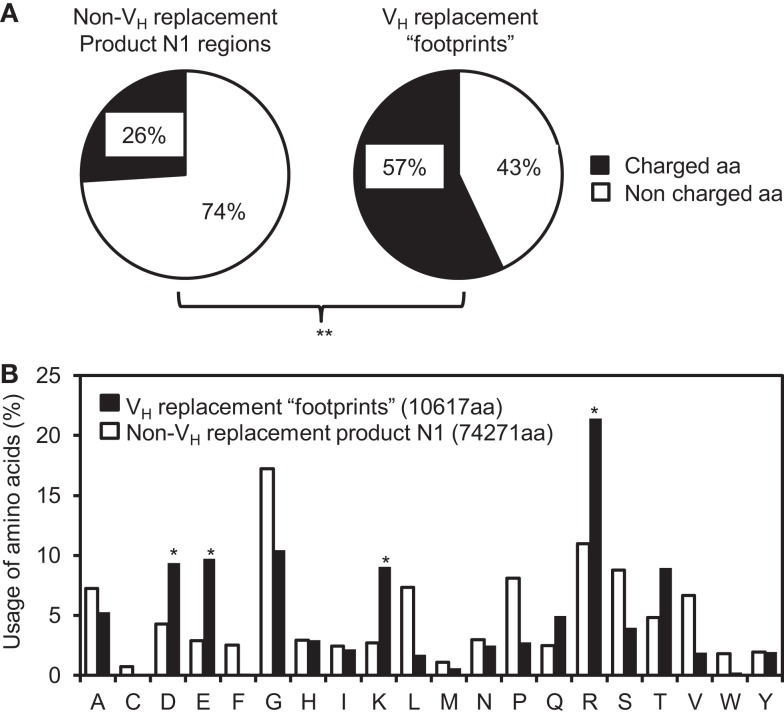
**V_H_ replacement footprints preferentially contribute charged amino acids into the IgH CDR3 regions**. **(A)** Frequencies of charged and uncharged amino acids (aa) in the N1 regions of non-V_H_ replacement products were compared with those encoded by the V_H_ replacement footprints. **(B)** The usages of different amino acids in the N1 regions of non-V_H_ replacement products (white bars) or encoded by the V_H_ replacement footprints (black bars) were analyzed and shown in the bar graph. The total number of amino acids analyzed for each population is indicated. Statistical significance was determined using a two-tailed Chi square test with Yate’s correction. *p < 0.05 is considered statistically significant. **p < 0.0001 is considered extremely statistically significant.

Our previous analysis showed that the V_H_ replacement footprints preferentially encoded charged amino acids in the IgH CDR3 regions (37, 45). This is likely predetermined by the conservation of amino acid sequence at the 3′ ends of V_H_ germline genes. Here, analysis of the amino acids encoded by the identified pentameric V_H_ replacement footprints in the 4417 V_H_ replacement products showed that 57% of them are charged amino acids. Such frequency is extremely statistically significantly higher than that in the N1 regions of non-V_H_ replacement products (25%) (Figure 5A, *p* < 0.0001, two-tailed Chi square test with Yates’ correction). Detailed analyses showed that the frequencies of K, R, D, and E residues encoded by the V_H_ replacement footprints are statistically significantly higher than their usage in the N1 regions of non-V_H_ replacement products (Figure 5B, *p* < 0.05, two-tailed Chi square test with Yates’ correction). These results confirmed our previous prediction that V_H_ replacement footprints preferentially contribute charged amino acids to the IgH CDR3 regions.

### V_H_ replacement products are positively selected during autoimmune or anti-viral responses

**Figure 6:**
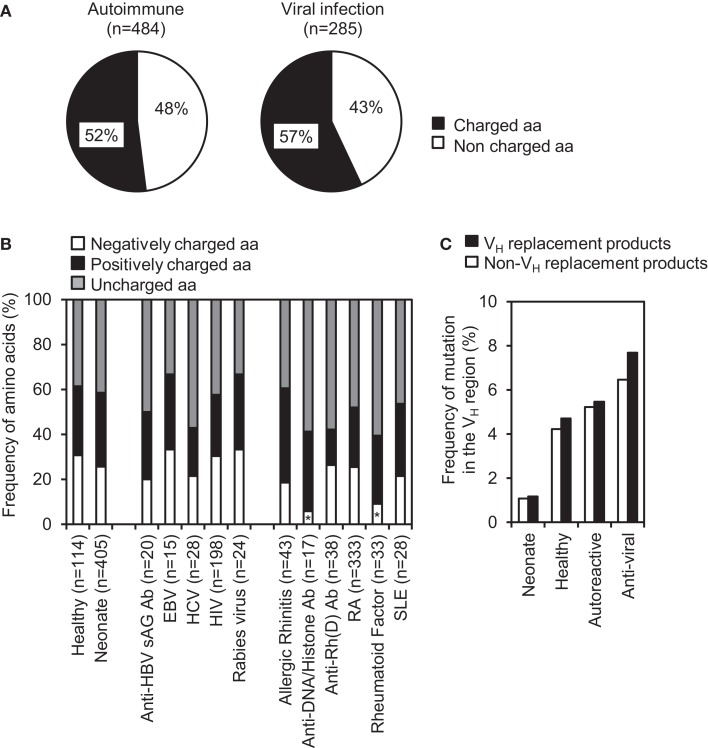
**V_H_ replacement footprints preferentially contribute charged amino acids into the CDR3 regions of IgH genes derived from autoimmune diseases and viral infections**. **(A)** Frequencies of charged and uncharged amino acids encoded by the V_H_ replacement footprints in IgH genes derived from autoimmune diseases and viral infections. *n*, Total number of amino acids analyzed in each subcategory. **(B)** Frequencies of negatively and positively charged residues encoded by the V_H_ replacement footprints in IgH genes derived from autoimmune diseases or viral infections. Statistical significance was determined using a two-tailed Chi square test with Yate’s correction. *p < 0.05 is considered statistically significant. **(C)** Comparison of overall somatic mutation rates (%) within V_H_ region of V_H_ replacement products versus non-V_H_ replacement products in different sub-categories.

Charged amino acids within IgH CDR3 are not well tolerated during Ab repertoire development, they are frequently found within the IgH CDR3 regions of autoreactive or anti-viral Abs, which may play important roles in binding charged self or viral antigens, respectively. Further analyses of V_H_ replacement products derived from different autoimmune diseases or viral infections showed that the identified V_H_ replacement footprints predominantly encode charged amino acids (Figure 6A). Detailed analyses showed that the identified V_H_ replacement footprints in IgH genes encoding anti-DNA/histone Abs or rheumatoid factors encoded significantly lower frequencies of negatively charged residues, including D, E, N, and Q residues (Figure 6B, *p* < 0.05, two-tailed Chi square test with Yates’ correction).

The identified V_H_ replacement products have similar mutation rate when compared with the non-V_H_ replacement product derived from healthy donors, patients with autoimmune diseases or viral infections (Figure 6C). As negative controls, V_H_ replacement products or non-V_H_ replacement products in neonatal IgH gene sequences have much lower mutation rates (Figure 6C). The accumulation of mutations within these V_H_ replacement products indicates that these enriched V_H_ replacement products in autoimmune diseases or viral infections had been positively selected.

## Discussion

In order to determine the distribution of V_H_ replacement products in these IgH genes and explore the biological significance of V_H_ replacement products in human antibody diversification and diseases, we developed a Java based computer program V_H_RFA to analyze large number of IgH gene sequences and to identify potential V_H_ replacement products (42). Previous analyses of the IgH gene repertoire have provided important insights regarding the developmental process and function of B lineage cells. Due to the tremendous diversity, the complete human IgH repertoire cannot be experimentally determined. Within the NCBI database, there are 61,851 human IgH gene sequences (May, 2012 version). The initial analysis of the V_H_, D_H_, and J_H_ gene usages in the 61,851 human IgH gene sequences provides a comprehensive view of the human IgH repertoire. In this dataset, the usage of every functional V_H_ germline gene was confirmed, although their usages differ dramatically.

Using the V_H_RFA program, we identified V_H_ replacement products and analyzed their distributions in the 39,438 unique IgH sequences. Based on the identification of pentameric V_H_ replacement footprint motifs within the V_H_–D_H_ junctions, 12.1% of the IgH genes can be assigned as potential V_H_ replacement products. These results confirmed our previous estimation that V_H_ replacement products contribute to the diversification of the human IgH repertoire. Interestingly, the frequencies of V_H_ replacement products in IgH genes using the V_H2–5_, V_H3–30_, V_H3-30-3_, V_H3–49_, V_H1–69_, and V_H3–34_ are statistically significantly higher than that in the overall IgH genes. In contrast, the frequency of V_H_ replacement products in IgH genes using the V_H6-1_ gene is statistically significantly lower than that in the overall IgH genes. Among the non-functional IgH genes, 18% of them contain the pentameric V_H_ replacement footprints and can be assigned as potential V_H_ replacement products. These results confirmed the prediction that V_H_ replacement is a random process that can generate both functional and non-functional IgH rearrangements. Moreover, the high frequency of V_H_ replacement products in non-functional IgH genes suggested that V_H_ replacement products were negatively selected during B-cell development. Based on this reasoning, the frequency of V_H_ replacement products in the non-functional IgH genes may represent the true frequency of V_H_ replacement during early stages of B-cell development, because these non-functional IgH rearrangements cannot encode BCRs and had not been selected during B-cell development.

Due to the location of the cRSS site, a short stretch of nucleotides has the potential to remain as a V_H_ replacement footprint at the V_H_–D_H_ junctions following the V_H_ replacement process (25, 37, 46). The leftover V_H_ replacement footprints will elongate the IgH CDR3 regions (25, 37, 46). Analyses of the identified 4788 V_H_ replacement products showed that the average CDR3 length of the identified V_H_ replacement products is 2.8 aa longer than that of non-V_H_ replacement products. Previously, it surprised us that the identified V_H_ replacement footprints preferentially encode charged amino acids within the IgH CDR3 regions (22, 37, 46). Recent analyses showed that the positions of the cRSS and high frequencies of charged amino acids encoded by the following nucleotides are highly conserved in IgH genes from different vertebrates (47). In the current study, 57% of the identified V_H_ replacement footprints encoded charged amino acids in the IgH CDR3 regions. Normally, charged amino acids within IgH CDR3 are not well tolerated during antibody repertoire development, probably due to charged residues may generate autoAbs. Indeed, our analysis revealed that V_H_ replacement products are significantly enriched in IgH genes derived from patients with different autoimmune diseases, including RA, allergic rhinitis, and SLE or in IgH genes encoding different autoAbs such as rheumatoid factor, anti-rhesus D antigen, and anti-acetylcholine receptor Abs. Our recent analyses of large number of mouse IgH genes also showed that the frequencies of V_H_ replacement products are enriched in IgH genes derived from autoimmune prone mice (48). These results suggested that V_H_ replacement products contribute to the generation of autoantibodies in both human and mouse.

Another important and interesting finding from this analysis of large number of IgH gene sequences is that the frequencies of V_H_ replacement products are significantly elevated in IgH genes derived from various viral infections, including HIV, HCV, and in IgH genes encoding Abs against HCV glycoprotein E2 or HBV surface antigens. Our recent studies showed that V_H_ replacement products are highly enriched in IgH genes encoding different subgroups of anti-HIV antibodies, especially in CD4i and PGT antibodies (49). These results suggested that V_H_ replacement products may contribute to the generation of anti-viral Abs. The majority of the V_H_ replacement footprints identified from anti-viral Abs also encode charged amino acids, which may be important for binding charged viral antigens. Moreover, the accumulation of mutations in these V_H_ replacement products indicated that these enriched V_H_ replacement products in patients with viral infections are positively selected during anti-viral responses. The identification of V_H_ replacement products in autoimmune diseases and anti-viral responses suggested a potential link between viral infections and the pathogenesis of autoimmune diseases. It has long been postulated that chronic viral infections contribute to autoimmunity. However, clear examples that Abs against viral antigens cross-react with self-antigens have only been found in a few cases (50, 51). Here, our results reveal a shared pattern of accumulation of V_H_ replacement products in IgH genes derived from autoimmune diseases and anti-viral responses.

V_H_ replacement was originally proposed as a receptor editing mechanism to change unwanted IgH genes that are either non-functional or encoding autoreactive Abs. The enrichment of V_H_ replacement products in IgH genes derived from autoimmune diseases or encoding autoAbs is particular puzzling. There are several possible mechanisms to explain this finding. First, we have recently shown that crosslinking cell surface BCRs induces V_H_ replacement in human immature B-cells (40). Thus, the levels of V_H_ replacement recombination might be induced in the immature B-cells during either the anti-viral immune response or autoimmune disease due to persistent antigen stimulation or chronic inflammation. In supporting of this assumption, the number of newly emigrated immature B-cells in the peripheral blood is increased during inflammatory response; and these mobilized immature B-cells may continue to undergo V_H_ replacement recombination ectopically. Second, the intrinsic feature of V_H_ replacement is elongating the IgH CDR3 with charged amino acid. V_H_ replacement products may frequently encode autoAbs and they are efficiently deleted during normal B-cell development. The observed elevated frequencies of V_H_ replacement products in different autoimmune diseases may reflect the defective negative selection in these diseased subjects. Moreover, ectopically occurred V_H_ replacement may bypass the stringent negative selection in the bone marrow and release V_H_ replacement products in the periphery. Last, due to the special features of V_H_ replacement products in generating IgH genes with long and charged CDR3, it is possible that V_H_ replacement products are positively selected by viral antigens during anti-viral responses to produce specific anti-viral Abs. In supporting of this notion, the identified potential V_H_ replacement products encoding anti-HIV antibodies all have very long CDR3 regions with multiple charged amino acid residues (49). The accumulated mutations within the V_H_ genes of the identified V_H_ replacement products in the current study also indicated the positive selection. However, the leftover V_H_ replacement products generated during a chronic viral infection may encode Abs that cross-react with self-antigens and later contribute to autoimmunity. In fact, many cell surface antigens and viral antigens are negatively charged, which may be a reason for the selection of V_H_ replacement products with long and charged CDR3 regions.

In our sequence based analysis, the assignment of V_H_ replacement is dependent on the identification of V_H_ replacement footprints within the V_H_–D_H_ junctions. Any deletion at the 3′ of V_H_ genes or the 5′ of V_H_ replacement footprint motifs during the primary or secondary IgH gene recombination, respectively, may destroy the pentameric V_H_ replacement footprints. Therefore, it is possible that the sequence analysis based study still under-estimates the frequency of V_H_ replacement products. Using the V_H_RFA program, we extended our analysis our V_H_ replacement products to include potential V_H_ replacement footprint motifs with different lengths. For examples, 33.9% of the IgH genes contain the tetrameric V_H_ replacement footprint motifs and 58.8% of IgH genes contain the trimeric V_H_ replacement footprint motifs. These results revealed a significant contribution of V_H_ replacement products to the IgH repertoire. Recent studies in mice carrying non-functional IgH genes on both IgH alleles demonstrated that V_H_ replacement occurs efficiently to generate almost normal numbers of B-cells with diversified IgH repertoires (52). However, only about 20% of the IgH gene sequences from this study contained residual V_H_ replacement footprints. Therefore, the majority IgH genes generated through V_H_ replacement recombination have no leftover V_H_ replacement footprints. Theoretically, 66% of IgH rearrangements will be out of reading frame and 44% of developing B-cells may carry non-functional IgH rearrangements on both alleles. If all of these B-cells are rescued by V_H_ replacement, a minimum of 44% of the IgH genes might be generated through V_H_ replacement recombination. Under this assumption, IgH genes containing the tetrameric or the trimeric V_H_ replacement footprint motifs at their N1 regions should also be considered as potential V_H_ replacement products.

Like any sequence based analysis program, the V_H_RFA program also has its limitation. Although sequence motifs assemble the V_H_ gene 3′ ending sequences can be identified in the N1 regions, such motifs can also be identified within the N2 regions at relative lower frequencies. Theoretically, V_H_ replacement can only leave footprint within the N1 region; the existence of V_H_ replacement footprint like motifs within the N2 regions can only be generated by random nucleotide addition. For IgH genes using the V_H6-1_ gene, which is the first V_H_ germline gene 5′ to the DH locus, there should have no V_H_ replacement footprint like motifs within the V_H_–D_H_ junctions, but the V_H_RFA program still identifies 7.56% of the sequences contains V_H_ replacement footprint like motifs within the V_H_–D_H_ junctions. We can only refer such motifs as the contribution of random nucleotide addition.

In summary, analyses of a large number of human IgH gene sequences from the NCBI database uncovered a significant contribution of V_H_ replacement products to human Ab repertoire, especially in IgH genes derived from autoimmune diseases or anti-viral responses. Understanding how V_H_ replacement is regulated and how V_H_ replacement products are positively or negatively selected during normal or diseased conditions will be the focus of future studies, because modulation of the level of V_H_ replacement may offer unique approaches to treat different human diseases.

## Conflict of Interest Statement

The authors declare that the research was conducted in the absence of any commercial or financial relationships that could be construed as a potential conflict of interest.

## Supplementary Material

The Supplementary Material for this article can be found online at http://www.frontiersin.org/Journal/10.3389/fimmu.2014.00345/abstract

## Abbreviations

aa, amino acid; cRSS, cryptic recombination signal sequence; EBV, Epstein–Barr virus; HBV, hepatitis virus B; HCV, hepatitis virus C; HIV, human immunodeficiency virus; RA, rheumatoid arthritis; RA, rheumatoid arthritis; RAG, recombination activating gene products; SLE, systemic lupus erythematosus; V_H_RFA, V_H_ replacement footprint analyzer.

## References

[1] Meffre E Casellas R Nussenzweig MC Antibody regulation of B cell development. Nat Immunol (2000) 1:379–85 10.1038/80816 11062496

[2] Rajewsky K Clonal selection and learning in the antibody system. Nature (1996) 381:751–8 10.1038/381751a0 8657279

[3] Tonegawa S Somatic generation of antibody diversity. Nature (1983) 302:575–81 10.1038/302575a0 6300689

[4] Oettinger MA Schatz DG Gorka C Baltimore D RAG-1 and RAG-2, adjacent genes that synergistically activate V(D)J recombination. Science (1990) 248:1517–23 10.1126/science.2360047 2360047

[5] Schatz DG Baltimore D Stable expression of immunoglobulin gene V(D)J recombinase activity by gene transfer into 3T3 fibroblasts. Cell (1988) 53:107–15 10.1016/0092-8674(88)90492-8 3349523

[6] Schatz DG Oettinger MA Baltimore D The V(D)J recombination activating gene, RAG-1. Cell (1989) 59:1035–48 10.1016/0092-8674(89)90760-5 2598259

[7] Lewis SM The mechanism of V(D)J joining: lessons from molecular, immunological, and comparative analyses. Adv Immunol (1994) 56:27–150 10.1016/S0065-2776(08)60450-2 8073949

[8] Ramsden DA Baetz K Wu GE Conservation of sequence in recombination signal sequence spacers. Nucleic Acids Res (1994) 22:1785–96 10.1093/nar/22.10.1785 8208601 PMC308075

[9] Swanson PC Desiderio S V(D)J recombination signal recognition: distinct, overlapping DNA-protein contacts in complexes containing RAG1 with and without RAG2. Immunity (1998) 9:115–25 10.1016/S1074-7613(00)80593-2 9697841

[10] ten Boekel E Melchers F Rolink AG Changes in the V(H) gene repertoire of developing precursor B lymphocytes in mouse bone marrow mediated by the pre-B cell receptor. Immunity (1997) 7:357–68 10.1016/S1074-7613(00)80357-X 9324356

[11] ten Boekel E Melchers F Rolink AG Precursor B cells showing H chain allelic inclusion display allelic exclusion at the level of pre-B cell receptor surface expression. Immunity (1998) 8:199–207 10.1016/S1074-7613(00)80472-0 9492001

[12] Alt FW Yancopoulos GD Blackwell TK Wood C Thomas E Boss M Ordered rearrangement of immunoglobulin heavy chain variable region segments. EMBO J (1984) 3:1209–19 6086308 10.1002/j.1460-2075.1984.tb01955.x PMC557501

[13] Burrows P LeJeune M Kearney JF Evidence that murine pre-B cells synthesise μ heavy chains but no light chains. Nature (1979) 280:838–40 10.1038/280838a0 112480

[14] Cappione AJ Pugh-Bernard AE Anolik JH Sanz I Lupus IgG VH4.34 antibodies bind to a 220-kDa glycoform of CD45/B220 on the surface of human B lymphocytes. J Immunol (2004) 172:4298–307 10.4049/jimmunol.172.7.4298 15034044

[15] Odendahl M Jacobi A Hansen A Feist E Hiepe F Burmester GR Disturbed peripheral B lymphocyte homeostasis in systemic lupus erythematosus. J Immunol (2000) 165:5970–9 10.4049/jimmunol.165.10.5970 11067960

[16] Pugh-Bernard AE Silverman GJ Cappione AJ Villano ME Ryan DH Insel RA Regulation of inherently autoreactive VH4-34 B cells in the maintenance of human B cell tolerance. J Clin Invest (2001) 108:1061–70 10.1172/JCI12462 11581307 PMC200949

[17] Bridges SL Lavelle JC Lee SK Byer S Schroeder HW CDR3 fingerprinting of immunoglobulin kappa light chains expressed in rheumatoid arthritis. Evidence of antigenic selection or dysregulation of gene rearrangement in B cells. Ann N Y Acad Sci (1997) 815:423–6 10.1111/j.1749-6632.1997.tb52093.x 9186688

[18] Bridges SL Lee SK Koopman WJ Schroeder HW Analysis of immunoglobulin gamma heavy chain expression in synovial tissue of a patient with rheumatoid arthritis. Arthritis Rheum (1993) 36:631–41 10.1002/art.1780360509 8489540

[19] Grimaldi CM Hicks R Diamond B B cell selection and susceptibility to autoimmunity. J Immunol (2005) 174:1775–81 10.4049/jimmunol.174.4.1775 15699102

[20] Rajewsky K Forster I Cumano A Evolutionary and somatic selection of the antibody repertoire in the mouse. Science (1987) 238:1088–94 10.1126/science.3317826 3317826

[21] Nemazee D Weigert M Revising B cell receptors. J Exp Med (2000) 191:1813–7 10.1084/jem.191.11.1813 10839798 PMC2213535

[22] Zhang Z Burrows PD Cooper MD The molecular basis and biological significance of VH replacement. Immunol Rev (2004) 197:231–42 10.1111/j.0105-2896.2004.0107.x 14962199

[23] Casellas R Shih TA Kleinewietfeld M Rakonjac J Nemazee D Rajewsky K Contribution of receptor editing to the antibody repertoire. Science (2001) 291:1541–4 10.1126/science.1056600 11222858

[24] Melamed D Benschop RJ Cambier JC Nemazee D Developmental regulation of B lymphocyte immune tolerance compartmentalizes clonal selection from receptor selection. Cell (1998) 92:173–82 10.1016/S0092-8674(00)80912-5 9458042

[25] Zhang Z VH replacement in mice and humans. Trends Immunol (2007) 28:132–7 10.1016/j.it.2007.01.003 17258935

[26] Kleinfield R Hardy RR Tarlinton D Dangl J Herzenberg LA Weigert M Recombination between an expressed immunoglobulin heavy-chain gene and a germline variable gene segment in a Ly 1+ B-cell lymphoma. Nature (1986) 322:843–6 10.1038/322843a0 3092106

[27] Reth M Gehrmann P Petrac E Wiese P A novel VH to VHDJH joining mechanism in heavy-chain-negative (null) pre-B cells results in heavy-chain production. Nature (1986) 322:840–2 10.1038/322840a0 3092105

[28] Usuda S Takemori T Matsuoka M Shirasawa T Yoshida K Mori A Immunoglobulin V gene replacement is caused by the intramolecular DNA deletion mechanism. EMBO J (1992) 11:611–8 1311252 10.1002/j.1460-2075.1992.tb05093.x PMC556493

[29] Chen C Nagy Z Prak EL Weigert M Immunoglobulin heavy chain gene replacement: a mechanism of receptor editing. Immunity (1995) 3:747–55 10.1016/1074-7613(95)90064-0 8777720

[30] Chen C Nagy Z Radic MZ Hardy RR Huszar D Camper SA The site and stage of anti-DNA B-cell deletion. Nature (1995) 373:252–5 10.1038/373252a0 7816141

[31] Chen C Prak EL Weigert M Editing disease-associated autoantibodies. Immunity (1997) 6:97–105 10.1016/S1074-7613(00)80673-1 9052841

[32] Cascalho M Ma A Lee S Masat L Wabl M A quasi-monoclonal mouse. Science (1996) 272:1649–52 10.1126/science.272.5268.1649 8658139

[33] Cascalho M Wong J Wabl M VH gene replacement in hyperselected B cells of the quasimonoclonal mouse. J Immunol (1997) 159:5795–801 9550375

[34] Lutz J Muller W Jack HM VH replacement rescues progenitor B cells with two nonproductive VDJ alleles. J Immunol (2006) 177:7007–14 10.4049/jimmunol.177.10.7007 17082616

[35] Davila M Liu F Cowell LG Lieberman AE Heikamp E Patel A Multiple, conserved cryptic recombination signals in VH gene segments: detection of cleavage products only in pro B cells. J Exp Med (2007) 204:3195–208 10.1084/jem.20071224 18056287 PMC2150985

[36] Watson LC Moffatt-Blue CS McDonald RZ Kompfner E it-Azzouzene D Nemazee D Paucity of V-D-D-J rearrangements and VH replacement events in lupus prone and nonautoimmune TdT-/- and TdT+/+ mice. J Immunol (2006) 177:1120–8 10.4049/jimmunol.177.2.1120 16818769

[37] Zhang Z Zemlin M Wang Y-H Munfus D Huye LE Findley HW Contribution of VH gene replacement to the primary B cell repertoire. Immunity (2003) 19:21–31 10.1016/S1074-7613(03)00170-5 12871636

[38] Sandel PC Monroe JG Negative selection of immature B cells by receptor editing or deletion is determined by site of antigen encounter. Immunity (1999) 10:289–99 10.1016/S1074-7613(00)80029-1 10204485

[39] Verkoczy L it-Azzouzene D Skog P Martensson A Lang J Duong B A role for nuclear factor kappa B/rel transcription factors in the regulation of the recombinase activator genes. Immunity (2005) 22:519–31 10.1016/j.immuni.2005.03.006 15845455 PMC3792720

[40] Liu J Lange MD Hong SY Xie W Xu K Huang L Regulation of VH replacement by B cell receptor-mediated signaling in human immature B cells. J Immunol (2013) 190:5559–66 10.4049/jimmunol.1102503 23630348 PMC3660396

[41] Radic M Zouali M Receptor editing, immune diversification and self-tolerance. Immunity (1996) 5:505–11 10.1016/S1074-7613(00)80266-6 8986711

[42] Huang L Lange MD Zhang Z VH replacement footprint analyzer-I, a Java-based computer program for analyses of immunoglobulin heavy chain genes and potential VH replacement products in human and mouse. Front Immunol (2014) 5:40 10.3389/fimmu.2014.00040 24575092 PMC3918983

[43] Zemlin M Bauer K Hummel M Pfeiffer S Devers S Zemlin C The diversity of rearranged immunoglobulin heavy chain variable region genes in peripheral blood B cells of preterm infants is restricted by short third complementarity-determining regions but not by limited gene segment usage. Blood (2001) 97:1511–3 10.1182/blood.V97.5.1511 11222402

[44] Brochet X Lefranc MP Giudicelli V IMGT/V-QUEST: the highly customized and integrated system for IG and TR standardized V-J and V-D-J sequence analysis. Nucleic Acids Res (2008) 36:W503–8 10.1093/nar/gkn316 18503082 PMC2447746

[45] Liu Y Fan R Zhou S Yu Z Zhang Z Potential contribution of VH gene replacement in immunity and disease. Ann N Y Acad Sci (2005) 1062:175–81 10.1196/annals.1358.020 16461800

[46] Zhang Z Wang YH Zemlin M Findley HW Bridges SL Burrows PD Molecular mechanism of serial VH gene replacement. Ann N Y Acad Sci (2003) 987:270–3 10.1111/j.1749-6632.2003.tb06060.x 12727651

[47] Sun Y Liu Z Li Z Lian Z Zhao Y Phylogenetic conservation of the 3’ cryptic recombination signal sequence (3’cRSS) in the VH genes of jawed vertebrates. Front Immunol (2012) 3:392 10.3389/fimmu.2012.00392 23267360 PMC3526766

[48] Huang L Lange MD Yu Y Li S Su K Zhang Z Contribution of VH replacement products in mouse antibody repertoire. PLoS One (2013) 8:e57877 10.1371/journal.pone.0057877 23469094 PMC3585286

[49] Liao H Guo JT Lange MD Fan R Zemlin M Su K Contribution of VH replacement products to the generation of anti-HIV antibodies. Clin Immunol (2013) 146:46–55 10.1016/j.clim.2012.11.003 23220404 PMC3649862

[50] Gross AJ Hochberg D Rand WM Thorley-Lawson DA EBV and systemic lupus erythematosus: a new perspective. J Immunol (2005) 174:6599–607 10.4049/jimmunol.174.11.6599 15905498

[51] McClain MT Heinlen LD Dennis GJ Roebuck J Harley JB James JA Early events in lupus humoral autoimmunity suggest initiation through molecular mimicry. Nat Med (2005) 11:85–9 10.1038/nm1167 15619631

[52] Koralov SB Novobrantseva TI Konigsmann J Ehlich A Rajewsky K Antibody repertoires generated by VH replacement and direct VH to JH joining. Immunity (2006) 25:43–53 10.1016/j.immuni.2006.04.016 16860756

