# Supplementary material for: Accumulation of VH Replacement Products in IgH Genes Derived from Autoimmune Diseases and Anti-Viral Responses in Human
Source: Front Immunol. 2014 Jul 22;5:345. doi: 10.3389/fimmu.2014.00345 (PMC4105631; doi:10.3389/fimmu.2014.00345)
Supplement: Supplementary file 1 [file Data_Sheet1.PDF]

Table S1. Human V_H genes containing the TACTGTG cRSS.

V_H Gene Name	3' sequence with cRSS	Sequences after cRSS	Functionality
IGHV1/OR15-1*01	TACTGTGCGAGA	CGAGA	noFuncSign
IGHV1/OR15-1*02	TACTGTGCGAGAGA	CGAGAGA	noFuncSign
IGHV1/OR15-1*03	TACTGTGCGAGAGA	CGAGAGA	noFuncSign
IGHV1/OR15-1*04	TACTGTGCGAGAGA	CGAGAGA	noFuncSign
IGHV1/OR15-2*01	TACTGTGCGAGAGA	CGAGAGA	noFuncSign
IGHV1/OR15-2*02	TACTGTGCGAGAGA	CGAGAGA	noFuncSign
IGHV1/OR15-2*03	TACTGTGCGAGAGA	CGAGAGA	noFuncSign
IGHV1/OR15-3*01	TACTGTGCGAGA	CGAGA	noFuncSign
IGHV1/OR15-3*02	TACTGTGCGAGAGA	CGAGAGA	noFuncSign
IGHV1/OR15-3*03	TACTGTGCGAGA	CGAGA	noFuncSign
IGHV1/OR15-4*01	TACTGTGCGAGA	CGAGA	noFuncSign
IGHV1/OR15-5*01	TACTGTGTGAGA	TGAGA	noFuncSign
IGHV1/OR15-5*02	TACTGTGTGAGA	TGAGA	noFuncSign
IGHV1/OR15-9*01	TACTGTGTGAGAGA	TGAGAGA	noFuncSign
IGHV1/OR21-1*01	TACTGTGTGAGAGA	TGAGAGA	noFuncSign
IGHV1-18*03	TACTGTGCGAGAGA	CGAGAGA	F
IGHV1-2*01	TACTGTGCGAGAGA	CGAGAGA	F
IGHV1-2*02	TACTGTGCGAGAGA	CGAGAGA	F
IGHV1-2*03	TACTGTGCGAGAGA	CGAGAGA	F
IGHV1-2*04	TACTGTGCGAGA	CGAGA	F
IGHV1-2*05	TACTGTGCGAGAGA	CGAGAGA	F
IGHV1-24*01	TACTGTGCAACAGA	CAACAGA	F
IGHV1-3*01	TACTGTGCGAGAGA	CGAGAGA	F
IGHV1-3*02	TACTGTGCGAGAGA	CGAGAGA	F
IGHV1-18*01	TACTGTGCGAGAGA	CGAGAGA	F
IGHV1-18*02			(F)
IGHV1-45*01	TACTGTGCAAGANA	CAAGANA	F
IGHV1-45*02	TACTGTGCAAGATA	CAAGATA	F
IGHV1-45*03	TACTGTGCAAGA	CAAGA	F
IGHV1-46*01	TACTGTGCGAGAGA	CGAGAGA	F
IGHV1-46*02	TACTGTGCGAGAGA	CGAGAGA	F
IGHV1-46*03	TACTGTGCTAGAGA	CTAGAGA	F
IGHV1-58*01	TACTGTGCGGCAGA	CGGCAGA	F
IGHV1-58*02	TACTGTGCGGCAGA	CGGCAGA	F
IGHV1-68*01	TACTGGGCAAGATA		P
IGHV1-69*01	TACTGTGCGAGAGA	CGAGAGA	F
IGHV1-69*02	TACTGTGCGAGA	CGAGA	F
IGHV1-69*03			F
IGHV1-69*04	TACTGTGCGAGAGA	CGAGAGA	F
IGHV1-69*05	TACTGTGCGAGA	CGAGA	F
IGHV1-69*06	TACTGTGCGAGAGA	CGAGAGA	F
IGHV1-69*07			F
IGHV1-69*08	TACTGTGCGAGAGA	CGAGAGA	F
IGHV1-69*09	TACTGTGCGAGAGA	CGAGAGA	F
IGHV1-69*10	TACTGTGCGAGAGA	CGAGAGA	F
IGHV1-69*11	TACTGTGCGAGAGA	CGAGAGA	F
IGHV1-69*12	TACTGTGCGAGAGA	CGAGAGA	F
IGHV1-69*13	TACTGTGCGAGAGA	CGAGAGA	(F)
IGHV1-8*01	TACTGTGCGAGAGG	CGAGAGG	F
IGHV1-8*02	TACTGTGCGAGAGG	CGAGAGG	F
IGHV1-c*01	TACTATGCAAGA		ORF
IGHV1-f*01	TACTGTGCAACA	CAACA	F
IGHV1-f*02			F
IGHV1-NL1*01	TACTGTGTGAGAGA	TGAGAGA	P

IGHV2/OR16-5*01	TACTGTGCATGGA	CATGGA	noFuncSign
IGHV2-5*01	TACTGTGCACACAGACC	CACACAGACC	F
IGHV2-5*02			F
IGHV2-5*03			F
IGHV2-5*04	TACTGTGTACGG	TACGG	F
IGHV2-5*05	TACTGTGCACACAGAC	CACACAGAC	F
IGHV2-5*06	TACTGTGCACACAGA	CACACAGA	F
IGHV2-5*07	TACTGTGTA	TA	F
IGHV2-5*08	TACTGTGCACACAGAC	CACACAGAC	F
IGHV2-5*09	TACTGTGCACACAGAC	CACACAGAC	F
IGHV2-5*10	TACTGTGCACGG	CACGG	F
IGHV2-10*01	TACTGTGCAAGGAGAC	CAAGGAGAC	P
IGHV2-26*01	TACTGTGCACGGATAC	CACGGATAC	F
IGHV2-70*01	TACTGTGCACGGATAC	CACGGATAC	F
IGHV2-70*02	TACTG		F
IGHV2-70*03	TACTG		F
IGHV2-70*04	TAC		F
IGHV2-70*05			F
IGHV2-70*06	TACTG		F
IGHV2-70*07	TACTG		F
IGHV2-70*08	TACTG		F
IGHV2-70*09	TACTGTGTACGG	TACGG	F
IGHV2-70*10	TACTGTGCACGGATAC	CACGGATAC	F
IGHV2-70*11	TACTGTGCACGGATAC	CACGGATAC	F
IGHV2-70*12	TACTGTGCACACAGAC	CACACAGAC	F
IGHV2-70*13	TATTGTGCACGGATAC		F
IGHV3/OR15-7*01	TACTGTGCTAGA	CTAGA	noFuncSign
IGHV3/OR15-7*02	TACTGTGCTAGA	CTAGA	noFuncSign
IGHV3/OR15-7*03	TACTGTGCTAGA	CTAGA	noFuncSign
IGHV3/OR15-7*05	TACTGTGCTAGAGA	CTAGAGA	noFuncSign
IGHV3/OR16-10*01	TACTGTGCAAGA	CAAGA	noFuncSign
IGHV3/OR16-10*02	TACTGTGCAAGA	CAAGA	noFuncSign
IGHV3/OR16-10*03	TACTGTGCAAGAGA	CAAGAGA	noFuncSign
IGHV3/OR16-12*01	TACTGTGCAAGA	CAAGA	noFuncSign
IGHV3/OR16-13*01	TACTGTACTAGA		noFuncSign
IGHV3/OR16-14*01	TACTGTACTAGA		noFuncSign
IGHV3/OR16-15*01	TACTGTGTGAGAAA	TGAGAAA	noFuncSign
IGHV3/OR16-15*02	TACTGTGTGAGA	TGAGA	noFuncSign
IGHV3/OR16-16*01	TACTGTGTGAGA	TGAGA	noFuncSign
IGHV3/OR16-6*02	TACTGTACCACAGG		noFuncSign
IGHV3/OR16-8*01	TACTGTGTGA	TGA	noFuncSign
IGHV3/OR16-8*02	TACTGTGTGAAACA	TGAAACA	noFuncSign
IGHV3/OR16-9*01	TACTGTGTGA	TGA	noFuncSign
IGHV3-9*01	TACTGTGCAAAAGATA	CAAAAGATA	F
IGHV3-9*02	TACTGTGCAAAAGATA	CAAAAGATA	F
IGHV3-11*01	TACTGTGCGAGAGA	CGAGAGA	F
IGHV3-11*03	TACTGTGCGAGA	CGAGA	F
IGHV3-11*04	TACTGTGCGAGAGA	CGAGAGA	F
IGHV3-13*01	TACTGTGCAAGAGA	CAAGAGA	F
IGHV3-13*02	TACTGTGCAAGAGA	CAAGAGA	F
IGHV3-13*03	TACTGTGCAAGA	CAAGA	F
IGHV3-13*04	TACTGTGCAAGAGA	CAAGAGA	F
IGHV3-15*01	TACTGTACCACAGA		F
IGHV3-15*02	TACTGTACCACAGA		F
IGHV3-15*03	TACTGTACCACAGA		F
IGHV3-15*04	TACTGTACCACAGA		F
IGHV3-15*05	TACTGTACCACAGA		F

IGHV3-15*06	TACTGTACCACAGA		F
IGHV3-15*07	TACTGTACCACAGA		F
IGHV3-15*08	TACTGTACCACAGG		F
IGHV3-16*01	TACTGTGTGAGAAA	TGAGAAA	ORF
IGHV3-16*02	TACTGTGTGAGAAA	TGAGAAA	ORF
IGHV3-19*01	TACTGTGTGAGAAA	TGAGAAA	P
IGHV3-20*01	CACTGTGCGAGAGA		F
IGHV3-21*01	TACTGTGCGAGAGA	CGAGAGA	F
IGHV3-21*02	TACTGTGCGAGAGA	CGAGAGA	F
IGHV3-21*03	TACTGTGCGAGAGA	CGAGAGA	F
IGHV3-21*04	TACTGTGCGAGAGA	CGAGAGA	F
IGHV3-22*01	TACTGTTCCAGAGA		P
IGHV3-22*02	TACTGTTCCAGAGA		P
IGHV3-23*01	TACTGTGCGAAAGA	CGAAAGA	F
IGHV3-23*02	TACTGTGCGAAAGA	CGAAAGA	F
IGHV3-23*03	TACTGTGCGAAA	CGAAA	F
IGHV3-23*04	TACTGTGCGAAAGA	CGAAAGA	F
IGHV3-23*05	TACTGTGCGAAA	CGAAA	F
IGHV3-25*01	TAGTGTACCAGAGA		P
IGHV3-25*02	TAGTGTACCAGAGA		P
IGHV3-25*03	TAGTGTACCAGA		P
IGHV3-25*04	TACTGTACCAGA		ORF
IGHV3-25*05	TAGTGTACCAGAGA		P
IGHV3-30*01	TACTGTGCGAGAGA	CGAGAGA	F
IGHV3-30*02	TACTGTGCGAAAGA	CGAAAGA	F
IGHV3-30*03	TACTGTGCGAGAGA	CGAGAGA	F
IGHV3-30*04	TACTGTGCGAGAGA	CGAGAGA	F
IGHV3-30*05	TACTGTGCGAGAGA	CGAGAGA	F
IGHV3-30*06	TACTGTGCGAGAGA	CGAGAGA	F
IGHV3-30*07	TACTGTGCGAGAGA	CGAGAGA	F
IGHV3-30*08	TACTGTGCGAGA	CGAGA	F
IGHV3-30*09	TACTGTGCGAGAGA	CGAGAGA	F
IGHV3-30*10	TACTGTGCGAGAGA	CGAGAGA	F
IGHV3-30*11	TACTGTGCGAGAGA	CGAGAGA	F
IGHV3-30*12	TACTGTGCGAGAGA	CGAGAGA	F
IGHV3-30*13	TACTGTGCGAGAGA	CGAGAGA	F
IGHV3-30*14	TACTGTGCGAGAGA	CGAGAGA	F
IGHV3-30*15	TACTGTGCGAGAGA	CGAGAGA	F
IGHV3-30*16	TACTGTGCGAGAGA	CGAGAGA	F
IGHV3-30*17	TACTGTGCGAGAGA	CGAGAGA	F
IGHV3-30*18	TACTGTGCGAAAGA	CGAAAGA	F
IGHV3-30*19	TACTGTGCGAGAGA	CGAGAGA	F
IGHV3-30-3*01	TACTGTGCGAGA	CGAGA	F
IGHV3-30-3*02	TACTGTGCGAAAGA	CGAAAGA	F
IGHV3-32*01	GGCTATACATAAGGTC		P
IGHV3-33*01	TACTGTGCGAGAGA	CGAGAGA	F
IGHV3-33*02	TACTGTGCGAGAGA	CGAGAGA	F
IGHV3-33*03	TACTGTGCGAAAGA	CGAAAGA	F
IGHV3-33*04	TACTGTGCGAGAGA	CGAGAGA	F
IGHV3-33*05	TACTGTGCGAGAGA	CGAGAGA	F
IGHV3-33*06	TACTGTGCGAAAGA	CGAAAGA	F
IGHV3-35*01	TACTGTGTGAGAAA	TGAGAAA	ORF
IGHV3-38*01	TACTGTGCCAGATATA	CCAGATATA	ORF
IGHV3-38*02	TACTGTGCCAGATATA	CCAGATATA	ORF
IGHV3-43*01	TACTGTGCAAAAGATA	CAAAAGATA	F
IGHV3-43*02	TACTGTGCAAAA	CAAAA	F
IGHV3-47*01	TATTGTGCAAGA		P

IGHV3-47*02	TATTGTGCAAGAGA		P
IGHV3-47*03	TATG		P
IGHV3-48*01	TACTGTGCGAGAGA	CGAGAGA	F
IGHV3-48*02	TACTGTGCGAGAGA	CGAGAGA	F
IGHV3-48*03	TACTGTGCGAGAGA	CGAGAGA	F
IGHV3-48*04	TACTGTGCGAGAGA	CGAGAGA	F
IGHV3-49*01	TACTGTACTAGAGA		F
IGHV3-49*02	TACTGTACTAGAGA		F
IGHV3-49*03	TACTGTACTAGAGA		F
IGHV3-49*04	TACTGTACTAGAGA		F
IGHV3-49*05	TACTGTACTAGAGA		F
IGHV3-52*01	TACTGTGTGAGAGG	TGAGAGG	P
IGHV3-52*02	TACTGTGTGAGA	TGAGA	P
IGHV3-52*03	TACTGTGTGAGA	TGAGA	P
IGHV3-53*01	TACTGTGCGAGAGA	CGAGAGA	F
IGHV3-53*02	TACTGTGCGAGA	CGAGA	F
IGHV3-53*03	TACTGTGCTAGGGA	CTAGGGA	F
IGHV3-53*04	TACTGTGCGAGAGA	CGAGAGA	F
IGHV3-54*01	TACTGTATGTGAGY		P
IGHV3-54*02	TACTGTATGTGAGG		P
IGHV3-54*04	TACTGTATGTGAGT		P
IGHV3-62*01	TACTGTGTGAAAGA	TGAAAGA	P
IGHV3-63*01	GGCTGTACATAAGGTT		P
IGHV3-63*02	GGCTGTACATAA		P
IGHV3-64*01	TACTGTGCGAGAGA	CGAGAGA	F
IGHV3-64*02	TACTGTGCGAGAGA	CGAGAGA	F
IGHV3-64*03	TACTGTGTGAAAGA	TGAAAGA	F
IGHV3-64*04	TACTGTGCGAGAGA	CGAGAGA	F
IGHV3-64*05	TACTGTGTGAAAGA	TGAAAGA	F
IGHV3-66*01	TACTGTGCGAGAGA	CGAGAGA	F
IGHV3-66*02	TACTGTGCGAGA	CGAGA	F
IGHV3-66*03	TACTGTGCGAGAGA	CGAGAGA	F
IGHV3-66*04	TACTGTGCGAGACA	CGAGACA	F
IGHV3-7*01	TACTGTGCGAGAGA	CGAGAGA	F
IGHV3-7*02	TACTGTGCGAGA	CGAGA	F
IGHV3-7*03	TACTGTGCGAGAGA	CGAGAGA	F
IGHV3-71*01	TACTGTGCGAGAGA	CGAGAGA	P
IGHV3-71*02	TACTGTGCGAGAGA	CGAGAGA	P
IGHV3-71*03	TACTGTGCGAGAGA	CGAGAGA	P
IGHV3-72*01	TACTGTGCTAGAGA	CTAGAGA	F
IGHV3-72*02			F
IGHV3-73*01	TACTGTACTAGACA		F
IGHV3-73*02	TACTGTACTAGACA		F
IGHV3-74*01	TACTGTGCAAGAGA	CAAGAGA	F
IGHV3-74*02	TACTGTGCAAGA	CAAGA	F
IGHV3-74*03	TACTGTGCAAGAGA	CAAGAGA	F
IGHV3-d*01	TACTGTAAGAAA		F
IGHV3-h*01	TACTGTGCGAGAGA	CGAGAGA	P
IGHV3-h*02	TACTGTGCGAGAGA	CGAGAGA	P
IGHV3-NL1*01	TACTGTGCGAAAGA	CGAAAGA	F
IGHV4/OR15-8*01	TACTGTGCGAGAGA	CGAGAGA	noFuncSign
IGHV4/OR15-8*02	TACTGTGCGAGAGA	CGAGAGA	noFuncSign
IGHV4/OR15-8*03	TACTGTGCGAGAGA	CGAGAGA	noFuncSign
IGHV4-4*01	TGCTGTGCGAGAGA		F
IGHV4-4*02	TACTGTGCGAGAGA	CGAGAGA	F
IGHV4-4*03	TACTG		F
IGHV4-4*04	TACTG		F

IGHV4-4*05	TACTG		F
IGHV4-4*06	TACTGTGCGAGAGA	CGAGAGA	F
IGHV4-4*07	TACTGTGCGAGAGA	CGAGAGA	F
IGHV4-28*01	TACTGTGCGAGAAA	CGAGAAA	F
IGHV4-28*02	TACTGTGCGAGAAA	CGAGAAA	F
IGHV4-28*03	TACTGTGCGAGAGA	CGAGAGA	F
IGHV4-28*04	TACTGTGCGAGA	CGAGA	F
IGHV4-28*05	TACTG		F
IGHV4-28*06	TACTGTGCGAGAAA	CGAGAAA	F
IGHV4-30-2*01	TACTGTGCCAGAGA	CCAGAGA	F
IGHV4-30-2*02	TACTGTGCG	CG	F
IGHV4-30-2*03	TACTGTGCGAGACA	CGAGACA	F
IGHV4-30-2*04	TACTGTGCGAGAGA	CGAGAGA	F
IGHV4-30-2*05	TACTGTGCCAGAGA	CCAGAGA	F
IGHV4-30-4*01	TACTGTGCCAGAGA	CCAGAGA	F
IGHV4-30-4*02	TACTGTGCCAGAGA	CCAGAGA	F
IGHV4-30-4*03	TACTG		F
IGHV4-30-4*04	TACTG		F
IGHV4-30-4*05	TACTGTGCCAGAGA	CCAGAGA	F
IGHV4-30-4*06	TACTGTGCCAGAGA	CCAGAGA	F
IGHV4-31*01	TACTGTGCGAGAGA	CGAGAGA	F
IGHV4-31*02	TACTGTGCGAGAGA	CGAGAGA	F
IGHV4-31*03	TACTGTGCGAGAGA	CGAGAGA	F
IGHV4-31*04	TACTGTGCG	CG	F
IGHV4-31*05	TACTGTGCG	CG	F
IGHV4-31*06	TACTG		F
IGHV4-31*07	TACTG		F
IGHV4-31*08	TACTG		F
IGHV4-31*09	TACTG		F
IGHV4-31*10	TACTGTGCGAGAGA	CGAGAGA	F
IGHV4-34*01	TACTGTGCGAGAGG	CGAGAGG	F
IGHV4-34*02	TACTGTGCGAGAGG	CGAGAGG	F
IGHV4-34*03	TACTG		F
IGHV4-34*04	TACTGTGCGAGAGG	CGAGAGG	F
IGHV4-34*05	TACTGTGCGAGAGG	CGAGAGG	F
IGHV4-34*06	TACTG		F
IGHV4-34*07	TACTG		F
IGHV4-34*08	TACTGTGCG	CG	F
IGHV4-34*09	TACTGTGCGAGAGA	CGAGAGA	F
IGHV4-34*10	TACTGTGCGAGATA	CGAGATA	F
IGHV4-34*11	TGCTGTGCGAGAGA		F
IGHV4-34*12	TACTGTGCGAGA	CGAGA	F
IGHV4-34*13	TACTGTGCGAGAGG	CGAGAGG	F
IGHV4-39*01	TACTGTGCGAGACA	CGAGACA	F
IGHV4-39*02	TACTGTGCGAGAGA	CGAGAGA	F
IGHV4-39*03	TACTG		F
IGHV4-39*04			F
IGHV4-39*05	TACTGTGCG	CG	F
IGHV4-39*06	TACTGTGCGAGAGA	CGAGAGA	F
IGHV4-39*07	TACTGTGCGAGAGA	CGAGAGA	F
IGHV4-55*01	TACTGTGCGAGATA	CGAGATA	P
IGHV4-55*02	TACTGTGCGAGATA	CGAGATA	P
IGHV4-55*03	TACTG		P
IGHV4-55*04	TACTG		P
IGHV4-55*05	TACTG		P
IGHV4-55*06	TACTG		P
IGHV4-55*07	TACT		P

IGHV4-55*08	TACTGTGCGAGAGA	CGAGAGA	P
IGHV4-55*09	TACTGTGCGAGAAA	CGAGAAA	P
IGHV4-59*01	TACTGTGCGAGAGA	CGAGAGA	F
IGHV4-59*02	TACTGTGCGAGAGA	CGAGAGA	F
IGHV4-59*03	TACTGTGCG	CG	F
IGHV4-59*04	TACTGTGCG	CG	F
IGHV4-59*05	TACTGTGCG	CG	F
IGHV4-59*06	TACTGTGCG	CG	F
IGHV4-59*07	TACTGTGCGAGA	CGAGA	F
IGHV4-59*08	TACTGTGCGAGACA	CGAGACA	F
IGHV4-59*09	TACTGTGCGAGAGG	CGAGAGG	F
IGHV4-59*10	TACTGTGCGAGATA	CGAGATA	F
IGHV4-61*01	TACTGTGCGAGAGA	CGAGAGA	F
IGHV4-61*02	TACTGTGCGAGAGA	CGAGAGA	F
IGHV4-61*03	TACTGTGCGAGAGA	CGAGAGA	F
IGHV4-61*04	TACTG		F
IGHV4-61*05	TACTGTGCGAGA	CGAGA	F
IGHV4-61*06	TACTGTGCCAGAGA	CCAGAGA	ORF
IGHV4-61*07	TACTGTGCGAGACA	CGAGACA	F
IGHV4-61*08	TACTGTGCGAGAGA	CGAGAGA	F
IGHV4-b*01	TACTGTGCGAGA	CGAGA	F
IGHV4-b*02	TACTGTGCGAGA	CGAGA	F
IGHV5-51*01	TACTGTGCGAGACA	CGAGACA	F
IGHV5-51*02	TACTGTGCGAGACA	CGAGACA	F
IGHV5-51*03	TACTGTGCGAGA	CGAGA	F
IGHV5-51*04	TACTGTGCGAGA	CGAGA	F
IGHV5-51*05			F
IGHV5-78*01	TATTGTGTGAGA		P
IGHV5-a*01	TACTGTGCGAGA	CGAGA	F
IGHV5-a*02	TACTGTGCGAGACA	CGAGACA	F
IGHV5-a*03	TACTGTGCGAGA	CGAGA	F
IGHV5-a*04	TACTGTGCGAGA	CGAGA	F
IGHV6-1*01	TACTGTGCAAGAGA	CAAGAGA	F
IGHV6-1*02	TACTGTGCAAGAGA	CAAGAGA	F
IGHV7-4-1*01	TACTGTGCGAGA	CGAGA	F
IGHV7-4-1*02	TACTGTGCGAGAGA	CGAGAGA	F
IGHV7-4-1*03			F
IGHV7-4-1*04	TACTGTGCGAGAGA	CGAGAGA	F
IGHV7-4-1*05	TACTGTGCGAGAGA	CGAGAGA	F
IGHV7-34-1*02	TACTGTGCGAAGTA	CGAAGTA	P
IGHV7-40*03	TACTGTGCGA	CGA	P
IGHV7-81*01	TACTGTGCGAGATA	CGAGATA	ORF

Table *S2*. Potential human V_H replacement footprint motifs with different length.

length(#)	10(1)	9(4)	8(7)	7(25)	6(40)	5(45)	4(41)	3(29)
potential VH replacement footprints	CACACAGACC	CACGGATAC	CACGGATA	CGAGAGA	CGAGAG	CGAGA	CGAG	CGA
		CACACAGAC	ACGGATAC	CAACAGA	GAGAGA	GAGAG	GAGA	GAG
		ACACAGACC	CACACAGA	CAAGANA	CAACAG	AGAGA	AGAG	AGA
		CAAAAGATA	ACACAGAC	CAAGATA	AACAGA	CAACA	CAAC	CAA
			CACAGACC	CTAGAGA	CAAGAN	AACAG	AACA	AAC
			CAAAAGAT	CGGCAGA	AAGANA	ACAGA	ACAG	ACA
			AAAAGATA	CGAGAGG	CAAGAT	CAAGA	CAGA	CAG
				CACGGAT	AAGATA	AAGAN	CAAG	AAG
				ACGGATA	CTAGAG	AGANA	AAGA	GAN
				CGGATAC	TAGAGA	AAGAT	AGAN	ANA
				CACACAG	CGGCAG	AGATA	GANA	GAT
				ACACAGA	GGCAGA	CTAGA	AGAT	ATA
				CACAGAC	GAGAGG	TAGAG	GATA	CTA
				ACAGACC	CACGGA	CGGCA	CTAG	TAG
				CAAGAGA	ACGGAT	GGCAG	TAGA	CGG
				CGAAAGA	CGGATA	GCAGA	CGGC	GGC
				CAAAAGA	GGATAC	AGAGG	GGCA	GCA
				AAAAGAT	CACACA	CACGG	GCAG	AGG
				AAAGATA	ACACAG	ACGGA	GAGG	CAC
				TGAAAGA	CACAGA	CGGAT	CACG	ACG
				CGAGACA	ACAGAC	GGATA	ACGG	GGA
				CGAGAAA	CAGACC	GATAC	CGGA	TAC
				CCAGAGA	CAAGAG	CACAC	GGAT	GAC
				CGAGATA	AAGAGA	ACACA	ATAC	ACC
					CGAAAG	CACAG	CACA	GAA
					GAAAGA	CAGAC	ACAC	AAA
					CAAAAG	AGACC	AGAC	TGA
					AAAAGA	TACGG	GACC	CCA
					AAAGAT	AAGAG	TACG	
					TGAAAG	CGAAA	CGAA	
					CGAGAC	GAAAG	GAAA	
					GAGACA	AAAGA	AAAG	
					CGAGAA	CAAAA	CAAA	
					GAGAAA	AAAAG	AAAA	
					CCAGAG	TGAAA	TGAA	
					CAGAGA	GAGAC	GACA	
					CGAGAT	AGACA	AGAA	
					GAGATA	GAGAA	CCAG	
						AGAAA		
						CCAGA		
						CAGAG		
						GAGAT		

Table S3. Identification of 4-mer V_H replacement footprint motifs in the 417 human IgH sequences.

Seq ID	VH	3'V-REGION	P3'V	N1	P5'D	DH	CDR3-IMGTAA
AF235859.1	IGHV6-1*01	tgtgcaaga		cgaccg		gggtcggg	ARRPGSGYYYGMDV
AF235818.1	IGHV1-69*06	tgtgcgaga		gaagcaaagtttgagaag		gctgccaaacc	AREAKFEKAAKPYYYYGMDV
AF235818.1	IGHV1-69*06	tgtgcgaga		gaagcaaagtttgagaag		gctgccaaacc	AREAKFEKAAKPYYYYGMDV
AF235818.1	IGHV1-69*06	tgtgcgaga		gaagcaaagtttgagaag		gctgccaaacc	AREAKFEKAAKPYYYYGMDV
AF235701.1	IGHV1-2*02	tgtgcgagaga	t	aggcat		tatggttcagggagttat	ARDRHYGSGSYYFDY
AF235900.1	IGHV3-48*03	tgtgcgagaga	t	tcggttgatctccag		gactacggtgactac	ARDSVDLQDYGDYERTLGDV
AF235820.1	IGHV3-21*01	tgtgcgagaga	tc	gggaccctgaggccctagtagc		ggatattgtagtagtaccagctgc	ARDRDPEALVADIVVVPAADNYYYYYGMDV
AF235820.1	IGHV3-21*01	tgtgcgagaga	tc	gggaccctgaggccctagtagc		ggatattgtagtagtaccagctgc	ARDRDPEALVADIVVVPAADNYYYYYGMDV
AF235820.1	IGHV3-21*01	tgtgcgagaga	tc	gggaccctgaggccctagtagc		ggatattgtagtagtaccagctgc	ARDRDPEALVADIVVVPAADNYYYYYGMDV
AF235903.1	IGHV3-33*01	tgtgcgagaga		cagac		agctgctgctgg	ARDRQLLLGYGMDV
AF235904.1	IGHV5-a*01	tgtgcgaga		actcacatgtc		tgtact	ARTHMSVLGWFDP
AF235720.1	IGHV5-51*05	t		actgtgcgagtccgagtccatcctc	c	gtggatacagctatggtt	CASPSPSSVDTAMVQIDI
AF235720.1	IGHV5-51*05	t		actgtgcgagtccgagtccatcctc	c	gtggatacagctatggtt	CASPSPSSVDTAMVQIDI
AF235823.1	IGHV3-11*01	tgtgcgagaga		caccctcacgaaatcacc		ttacgatttttggagtggttattat	ARDTLTKSPYDFWSGYYGLTYYYYGMDV
AF235823.1	IGHV3-11*01	tgtgcgagaga		caccctcacgaaatcacc		ttacgatttttggagtggttattat	ARDTLTKSPYDFWSGYYGLTYYYYGMDV
AF235656.1	IGHV3-15*01	tgtaccaca		tacgtagtggaac		ctatggttcgggg	TTYVVEPMVRGDYGMEV
AF235826.1	IGHV1-69*02	tgtgcgaga		gataaac		tggatacagctatggttac	ARDKLDTAMVTGTSYFDY
AF235856.1	IGHV6-1*01	tgtgcgagaga	tct	agtgaggc		attacgatattttgactggtt	ARDLVRHYDILTGSQDGMDV
AF235857.1	IGHV3-23*01	tgtgcgaaaga	t	gaagaggag		tattgtggtagaaccagctgct	AKDEEEYCGRTSCFCMDV
AF235857.1	IGHV3-23*01	tgtgcgaaaga	t	gaagaggag		tattgtggtagaaccagctgct	AKDEEEYCGRTSCFCMDV
AF235601.1	IGHV1-18*01	tgtgcgagaga		cgacggacgggcggcgg		attgtagtggtggtagctgctactcc	ARDDGRAADCSGGSCYSDY
AF235601.1	IGHV1-18*01	tgtgcgagaga		cgacggacgggcggcgg		attgtagtggtggtagctgctactcc	ARDDGRAADCSGGSCYSDY
AF235601.1	IGHV1-18*01	tgtgcgagaga		cgacggacgggcggcgg		attgtagtggtggtagctgctactcc	ARDDGRAADCSGGSCYSDY
AF235860.1	IGHV5-51*01	tgtgcgagac		tttggtgggggatcgattacgacctaaaa		tacaactgg	ARLWWGIDYDLKYNWLDP
AF235860.1	IGHV5-51*01	tgtgcgagac		tttggtgggggatcgattacgacctaaaa		tacaactgg	ARLWWGIDYDLKYNWLDP
AF235860.1	IGHV5-51*01	tgtgcgagac		tttggtgggggatcgattacgacctaaaa		tacaactgg	ARLWWGIDYDLKYNWLDP
AF235860.1	IGHV5-51*01	tgtgcgagac		tttggtgggggatcgattacgacctaaaa		tacaactgg	ARLWWGIDYDLKYNWLDP
AF235556.1	IGHV3-48*02	tgtgcgagag		tcggtatagcagctcgtcc		gtatagtgggagctact	ARVGIAARPYSGSYYY
AF235901.1	IGHV2-5*01	tgtgcacacagac		tgcaaacac		tggctac	AHRLQTLATRRAFDP
AF235901.1	IGHV2-5*01	tgtgcacacagac		tgcaaacac		tggctac	AHRLQTLATRRAFDP
AF235901.1	IGHV2-5*01	tgtgcacacagac		tgcaaacac		tggctac	AHRLQTLATRRAFDP
AF235609.1	IGHV3-33*05	tgtgcgaga		agagggccaatcc		atatcagcagctgg	ARRGPIHISSWYYYYYGMDV
AF235664.1	IGHV3-74*01	tgtgcgaga		tggggggatgg		atagtgggagctactac	ARWGMDSGSYYDTVEAFDI
AF235766.1	IGHV3-30*03	tgtgcga		aacagtggacgc		atattgtgg	AKQWTHIVVFDI
AF235713.1	IGHV5-51*01	tgtgcga		ttcaacgacccgcc		gcagcagctggt	AIQRPAAAAGSSDAFDI
AF235713.1	IGHV5-51*01	tgtgcga		ttcaacgacccgcc		gcagcagctggt	AIQRPAAAAGSSDAFDI
AF235891.1	IGHV3-21*01	tgtgcgaaag		gagggtcca		ggggttgggagtggctgg	AKGGSRGWEWLEHFDY
AF235871.1	IGHV5-a*01	tgtgcga		agcaaggtagggatcaac		gtgttggacctcgtc	AKQGRDQRVGPRRWFTAMDV

AF235871.1	IGHV5-a*01	tgtgcga		agcaaggtagggatcaac		gtgttggacctcgtc	AKQGRDQRVGPRRWFTAMDV
AF235871.1	IGHV5-a*01	tgtgcga		agcaaggtagggatcaac		gtgttggacctcgtc	AKQGRDQRVGPRRWFTAMDV
AF235871.1	IGHV5-a*01	tgtgcga		agcaaggtagggatcaac		gtgttggacctcgtc	AKQGRDQRVGPRRWFTAMDV
AF235654.1	IGHV4-59*07	tgtgcga		aaagtagca		gtagaccaggct	AKSSSRPGSPMDV
AF235634.1	IGHV5-51*01	tgtgcgagac		ccgcgaataccgtcatcgaa		agcagctggt	ARPANTVIESSWYAFDI
AF235634.1	IGHV5-51*01	tgtgcgagac		ccgcgaataccgtcatcgaa		agcagctggt	ARPANTVIESSWYAFDI
AF235634.1	IGHV5-51*01	tgtgcgagac		ccgcgaataccgtcatcgaa		agcagctggt	ARPANTVIESSWYAFDI
AF235806.1	IGHV3-15*01	tgt		cattcggggggtagacc		gtatagcagtggctggt	HSGGRPYSSGWSPKWYYGMDV
AF235806.1	IGHV3-15*01	tgt		cattcggggggtagacc		gtatagcagtggctggt	HSGGRPYSSGWSPKWYYGMDV
AF235848.1	IGHV3-30-3*01	tgtgcgaga		gtcgga		gggccttcctac	ARVGGPSYYGMDL
AF235787.1	IGHV3-23*01	tgtgcgaaaga	tc	aacctcgaaag		gcagcagctggta	AKDQPRKAAAGMYYYGMDV
AF235577.1	IGHV3-21*01	tgtgcgagag		gaca		agcagcagctgg	ARGQAAAGAEYFQH
AF235747.1	IGHV1-2*02	tgtgtgagag		gagg		ggggga	VRGGGDDAFDI
AF235850.1	IGHV5-51*01	tgtgcgagac		gagggatcaac		gaatactttgac	ARRGINEYFDL
AF235850.1	IGHV5-51*01	tgtgcgagac		gagggatcaac		gaatactttgac	ARRGINEYFDL
AF235850.1	IGHV5-51*01	tgtgcgagac		gagggatcaac		gaatactttgac	ARRGINEYFDL
AF235574.1	IGHV4-59*07	tgtgcgaga		cgaaat		tattactatgatagtagtggt	ARRNYYYDSSGPDAFDI
AF235740.1	IGHV1-69*06	tgtgcgaga		gatgccgccccacatg		attttgactggttat	ARDAAPHDFDWLSRDFDY
AF235726.1	IGHV1-69*06	tgtgcg		gggagaggagagtat		ggctatagcagcagctgg	AGRGEYGYSSSWFDY
AF235726.1	IGHV1-69*06	tgtgcg		gggagaggagagtat		ggctatagcagcagctgg	AGRGEYGYSSSWFDY
AF235576.1	IGHV3-30*08	tgtgcgagaga	t	gagggtcaa		atagcagtgg	ARDEGSNSSGDY
AF235607.1	IGHV3-33*05	tgtgcgag		gaccc		atagtgggagctact	ARTHSGSYSVAFDI
AF235644.1	IGHV1-2*02	tgtgcgagaga	t	ttagggt		gtagtaccagctgc	ARDLGCSTSCPYYYGMDV
AF235869.1	IGHV2-70*10	tgtgc		cagaca		atattgtggtggtgactgct	ARQYCGGDCCSDY
AF235869.1	IGHV2-70*10	tgtgc		cagaca		atattgtggtggtgactgct	ARQYCGGDCCSDY
AF235878.1	IGHV2-70*10	tgtgcacggatac	g	ggaggt		ctggaactac	ARIREVWNYRYDYYGMDV
AF235649.1	IGHV1-2*02	tgtgcgagaga		gaggggtcccgaggcgga		actaactgct	ARERGPEAELTAYDI
AF235649.1	IGHV1-2*02	tgtgcgagaga		gaggggtcccgaggcgga		actaactgct	ARERGPEAELTAYDI
AF235649.1	IGHV1-2*02	tgtgcgagaga		gaggggtcccgaggcgga		actaactgct	ARERGPEAELTAYDI
AF235649.1	IGHV1-2*02	tgtgcgagaga		gaggggtcccgaggcgga		actaactgct	ARERGPEAELTAYDI
AF235705.1	IGHV5-a*01	tgtgcgag		ggatccc		ggatacagctatggt	ARDPGYSYGYYYYGMDV
AF235809.1	IGHV4-39*07	tgtgcga		caaaatc	c	gtattacgatattttgactggttatt	ATKSVLRYFDWLLPSYYYYYGMDV
AF235800.1	IGHV3-23*01	tgtgcg		ccataccggggg	ac	gtattactatgatagtagtggttattac	APYRGTYYYDSSGYYADYYGMDV
AF235610.1	IGHV3-30-3*01	tgtgcgaga		gatgaaag		tagcagtggctgg	ARDESSSGWYWYFDL
AF235613.1	IGHV2-5*01	tgtgcac		ggacccagccca		ggtatagtgggagct	ARTQPRYSGSYYFDY
AF235613.1	IGHV2-5*01	tgtgcac		ggacccagccca		ggtatagtgggagct	ARTQPRYSGSYYFDY
AF235541.1	IGHV3-48*03	tgtgcgagaga	tc	gacgcgaccggat		taactgggga	ARDRRDRINWGYYYGMDV
AF235541.1	IGHV3-48*03	tgtgcgagaga	tc	gacgcgaccggat		taactgggga	ARDRRDRINWGYYYGMDV
AF235678.1	IGHV5-51*01	tgtgcgagaca	t	actcaag		gcagcagctggtac	ARHTQGSSWYYRY
AF235727.1	IGHV5-51*01	tgtgcgagaca	t	cggc		attacgatttttggagt	ARHRHYDFWSTYYYMDV
AF235663.1	IGHV5-a*01	tgtgcgaga		ctggaggtgtggga		agcagctcgtcc	ARLEVWEAARPPYWYFDL

AF235595.1	IGHV3-30*08	tgtgcgaga		tccggacccccc		aactgggga	ARSGPPNWGDYYYGMDV
AF235595.1	IGHV3-30*08	tgtgcgaga		tccggacccccc		aactgggga	ARSGPPNWGDYYYGMDV
AF235919.1	IGHV3-30*03	tgtgcgaaag		catacc		gtatagccttggctggtac	AKAYPYSLGWYGAYYYGLDV
AF235554.1	IGHV1-69*01	tgtgcgagag		cgagggtcata		tggagtggcta	ARARGHMEWLHAFDI
AF235554.1	IGHV1-69*01	tgtgcgagag		cgagggtcata		tggagtggcta	ARARGHMEWLHAFDI
AF235758.1	IGHV2-70*01	tgtgcacggata		agggccctagacgta		aactgggga	ARIRALDVNWGGWYFDL
AF235758.1	IGHV2-70*01	tgtgcacggata		agggccctagacgta		aactgggga	ARIRALDVNWGGWYFDL
AF235544.1	IGHV3-66*01	tgtgcgagaga	tc	gagac		tacgatttttggagtggtt	ARDRDYDFWSGYAFDI
AF235692.1	IGHV3-33*01	tgtgcgagaga		gggggagattgat		catattgtggtggtgactgctatccc	AREGEIDHIVVVTAIPNWFDP
AF235625.1	IGHV5-51*01	tgtgcgag		tggcggaagg		atagtgggagctac	ASGGRIVGATDAFDI
AF235700.1	IGHV4-59*07	tgtgcgaga		ggaggtgaatgg		atggttcagggagtta	ARGGEWMVQGVIDY
AF235700.1	IGHV4-59*07	tgtgcgaga		ggaggtgaatgg		atggttcagggagtta	ARGGEWMVQGVIDY
AF235916.1	IGHV3-33*05	tgtgcga		aggcaa		gtagtggtggta	AKASSGGTKYGMDV
AF235724.1	IGHV5-a*01	tgtgcgaga		aacgggg		acgatttttggagtggttatttttcc	ARNGDDFWSGYFSLDY
AF235738.1	IGHV3-23*01	tgtgcgagaga	tc	cgaccctcgggaag		tacgatttttggagtggttattatacc	ARDPTLGKYDFWSGYYTGTTYYYYYYMDV
AF235764.1	IGHV1-3*01	tgtgcgagag		cgaga	ct	aggatattgtagtggtggtagctgctactcc	ARARLGYCSGGSCYSGGFDY
AF235508.1	IGHV5-51*01	tgtgcgag		gacatcgactct	c	gggtatagcagtggctggt	ARTSTLGYSSGWYY
AF235536.1	IGHV1-3*01	tgtgcgagaga		gcacatca	t	aggatattgtactaatggtgtatgc	AREHIIGYCTNGVCHDAFDI
AF235698.1	IGHV3-30*03	tgtgcgaaaga	tc	cgagg		ataactggaactac	AKDPRITGTTFGDYYYYGMDV
AF235698.1	IGHV3-30*03	tgtgcgaaaga	tc	cgagg		ataactggaactac	AKDPRITGTTFGDYYYYGMDV
AF235797.1	IGHV1-46*01	tgtgcgagaga		cggcggtcc		tgggtggt	ARDGGPGWFYRPHYYYYGMDV
AF235793.1	IGHV1-69*02	tgtgcgaga		gatctcacttacgggc		attttgactggtta	ARDLTYGHFDWLPPHYYYYYGMDV
AF235897.1	IGHV3-21*01	tgtgcgaga		tcaacggcatca		tacggtgactac	ARSTASYGDYDNWFDP
AF235897.1	IGHV3-21*01	tgtgcgaga		tcaacggcatca		tacggtgactac	ARSTASYGDYDNWFDP
AF235897.1	IGHV3-21*01	tgtgcgaga		tcaacggcatca		tacggtgactac	ARSTASYGDYDNWFDP
AF235796.1	IGHV3-30*03	tgtgcgaaaga	tc	ctacgggaaccacaaacttatctcccttagggcg		agcagcagct	AKDPTGTTNLSPLGRAAAYVYYYYYGMDV
AF235796.1	IGHV3-30*03	tgtgcgaaaga	tc	ctacgggaaccacaaacttatctcccttagggcg		agcagcagct	AKDPTGTTNLSPLGRAAAYVYYYYYGMDV
AF235796.1	IGHV3-30*03	tgtgcgaaaga	tc	ctacgggaaccacaaacttatctcccttagggcg		agcagcagct	AKDPTGTTNLSPLGRAAAYVYYYYYGMDV
AF235796.1	IGHV3-30*03	tgtgcgaaaga	tc	ctacgggaaccacaaacttatctcccttagggcg		agcagcagct	AKDPTGTTNLSPLGRAAAYVYYYYYGMDV
AF235566.1	IGHV5-51*01	tgtgcgagac		tcatcgaagggggg		gatattttgactggttattat	ARLIEGGDILTGYYRGFDY
AF235894.1	IGHV3-64*05	tgcgtgaaaga	t	gcacatcc		tagtgggagctac	VKDAHPSGSYGWLDP
AF235686.1	IGHV5-51*01	tgtgcgagac		tctgggagg		gcagctcg	ARLWEGSSDAFDI
AF235588.1	IGHV4-59*08	tgtgcga		cccatcggat		taactgggga	ATHRINWGFDY
AF235593.1	IGHV1-2*04	tgtgcgaga		gatcgag		gcagctcg	ARDRGSSFDY
AF235907.1	IGHV5-51*01	tgtg		tgcgagacagctcg		tacagctatggtt	VRDSSYSYGLSNLYYYGMDV
AF235907.1	IGHV5-51*01	tgtg		tgcgagacagctcg		tacagctatggtt	VRDSSYSYGLSNLYYYGMDV
AF235531.1	IGHV3-48*01	tgtgcgagaga		cgaa		aacc	ARDENLRH
AF235535.1	IGHV3-30*08	tgtgcgagaga		cgaa		aacc	ARDENLRH
AF235842.1	IGHV3-23*01	tgtgcgaaaga	t	ttcccagacgagcccgg		gtaccagctgctatac	AKDFPDEPGYQLLYGSLDY
AF235842.1	IGHV3-23*01	tgtgcgaaaga	t	ttcccagacgagcccgg		gtaccagctgctatac	AKDFPDEPGYQLLYGSLDY
AF235842.1	IGHV3-23*01	tgtgcgaaaga	t	ttcccagacgagcccgg		gtaccagctgctatac	AKDFPDEPGYQLLYGSLDY

AF235841.1	IGHV3-23*01	tgtgcga		gaccac		gtagccctagcagc	ARPRSPSSRAFYYGMDV
AF235615.1	IGHV5-51*01	tgtgcgag		ccctccggcg	g	ctaactgggga	ASPPAANWGDAFDI
AF235794.1	IGHV3-23*01	tgtgcgaaaga		ccctcgggatcgct		ttactatggttcggggagtta	AKDPRDRFTMVRGVTPGFDY
AF235812.1	IGHV5-a*01	tgtgcgag		ggccgaaatcttatccgg		agcagtggc	ARAEILSGAVAPRDY
AF235717.1	IGHV1-69*01	tgtgcgagaga		cggaact	ctc	gagtatagcagctcgtc	ARDGTLEYSSSSQPGDY
AF235659.1	IGHV2-5*01	tgtgcac		gcgatcccgacac		ctacggtggta	ARDPDTYGGNYFDY
AF235659.1	IGHV2-5*01	tgtgcac		gcgatcccgacac		ctacggtggta	ARDPDTYGGNYFDY
AF235875.1	IGHV5-a*01	tgtgcgaga		cagcgaa		gtataggagtag	ARQRSIGVGYGMDV
AF235716.1	IGHV2-70*01	tgtgcacggat		gaggcctcc		cttat	ARMRPPLYYFDY
AF235718.1	IGHV5-51*01	tgtgcgaga		ttcgggcatatatctcc	tca	tgactacagtaacta	ARFGHISPHDYSNYYWYFDL
AF235815.1	IGHV3-33*01	tgtgcgagaga	t	gtcttgatggggat		ttactatgatagtagtggttattact	ARDVLMGIYYDSSGYYLGSDYYYGMDV
AF235709.1	IGHV1-18*01	tgtgc		cacgaccgga	ct	aggatattgttatggtgg	ATTGLGYCYGGLAATLYYGMDV
AF235709.1	IGHV1-18*01	tgtgc		cacgaccgga	ct	aggatattgttatggtgg	ATTGLGYCYGGLAATLYYGMDV
AF235709.1	IGHV1-18*01	tgtgc		cacgaccgga	ct	aggatattgttatggtgg	ATTGLGYCYGGLAATLYYGMDV
AF235863.1	IGHV3-72*01	tgtgctagaga	t	ggatttc		acagtaactac	ARDGFHSNYSGNYYYYGMDV
AF235657.1	IGHV5-51*01	tgtgcgagac		gagaacaacc		tgggacccact	ARREQPGTHLNY
AF235657.1	IGHV5-51*01	tgtgcgagac		gagaacaacc		tgggacccact	ARREQPGTHLNY
AF235657.1	IGHV5-51*01	tgtgcgagac		gagaacaacc		tgggacccact	ARREQPGTHLNY
AF235626.1	IGHV3-21*01	tgtggga		aagaggacc		ggagttatta	GKEDRSYYDY
AF235626.1	IGHV3-21*01	tgtggga		aagaggacc		ggagttatta	GKEDRSYYDY
AF235626.1	IGHV3-21*01	tgtggga		aagaggacc		ggagttatta	GKEDRSYYDY
AF235565.1	IGHV3-23*01	tgt		accacagacccggccttgaggacctc		actgctggggt	TTDPALRTSLLGSFDY
AF235565.1	IGHV3-23*01	tgt		accacagacccggccttgaggacctc		actgctggggt	TTDPALRTSLLGSFDY
AF235565.1	IGHV3-23*01	tgt		accacagacccggccttgaggacctc		actgctggggt	TTDPALRTSLLGSFDY
AF235565.1	IGHV3-23*01	tgt		accacagacccggccttgaggacctc		actgctggggt	TTDPALRTSLLGSFDY
AF235795.1	IGHV3-30*03	tgtgcga		agagttt		tgaaggtagtgg	AKSFEGSGNWFDP
AF235775.1	IGHV4-30-2*05	tgtgcgaga		aggttcgga		gatattttgactggttatt	ARRFGDILTGYYAFDI
AF235829.1	IGHV3-23*01	tgtgcgaa		gaggaagttg		ggatataatggctacga	AKRKLGYNGYEGGNWFDP
AF235697.1	IGHV3-48*03	tgtgcga		taacggcc	ac	gtattacgatttttggagtggttattatacc	AITATYYDFWSGYYTGISDDAFDI
AF235697.1	IGHV3-48*03	tgtgcga		taacggcc	ac	gtattacgatttttggagtggttattatacc	AITATYYDFWSGYYTGISDDAFDI
AF235599.1	IGHV3-33*01	tgtgcgag		gccctcaaat		tattgtactaatggtgtatgctat	ARPSNYCTNGVCYNWFDP
AF235590.1	IGHV3-66*01	tgtgcgag		gggcgagct		ggggatttatg	ARGELGIYDWFDP
AF235853.1	IGHV3-30*03	tgtgcgaggga	t	agcgggat		tgggggctac	ARDSGIGGYGDVYDI
AF235693.1	IGHV2-5*08	tgtgcacaca		tcaaacg		tttggagtggttaata	AHIKRLEWLIGAFDI

*One sequence with different potential 4-mer of V_H replacement footprints is included in a box.

Table S4. Identification of V_H replacement products in IgH genes derived from different keyword subcategories.

Accession #	V_H Gene	V_H	P	N1[a]	P	D_H	CDR3 (aa)[b]
**Healthy**							
DQ926090.1	IGHV1-2*02	tgtgcgagaga	t	ttctacgg		gaccggttacta	ARDFYGTGYYFDS
EF542564.1	IGHV3-53*01	tgt		ttgagacttactgatca		gagcatagc	LRLTDQSIADY
AF015143.1	IGHV6-1*01	c		tgtgcaaagaacatca		aactgggg	CKEHQTGGLTS
EF177950.1	IGHV3-48*02	tgtgcgagaga	t	tgtcggcagggc	ac	gtattactatgatagtagtggttatta	ARDCRQGTYYYDSSGYYNDAFDI
EF542570.1	IGHV3-72*01	tgtgct		tgcacctcacaggag		gactaccgatgggg	ACTSQEDYRWGLDDMAV
EF542562.1	IGHV3-23*01	tgtgcgaaag		tgaggcagctatg		ggtcatggttgct	AKVRQLWVMVAGALDI
EF178072.1	IGHV4-4*02	tgtgcgagaga	t	tatagagcatccccgaga		accgcta	ARDYRASPRTANDY
EF178072.1	IGHV4-4*02	tgtgcgagaga	t	tatagagcatccccgaga		accgcta	ARDYRASPRTANDY
EF177982.1	IGHV4-39*01	tgtgcgag		tagcagatttatt		tattactatgat	ASSRFIYYYDDQYYFDY
EF178134.1	IGHV3-7*03	tgtgcga		tcgagaggtctggagg		tagaaatgactggcac	AIERSGGRNDWHGGY
EF542667.1	IGHV1-2*02	tgtgcg		gggacgggtgtactagaaccgacagccaggacctctcctat		gtatagcagcagctggtac	AGTGVLEPTARTSPMYSSSWYGWFDP
EF178031.1	IGHV3-23*01	tgtgcgaaag		gctatacggg		agcagttggta	AKGYTGAVGIGLDY
DQ926142.1	IGHV3-33*01	tgtacgagaga	t	gacgatacaaa		tagcagatactcc	TRDDDTNSRYSRLY
EF542577.1	IGHV3-30*19	tgtgcgagaga	tc	gcgaaac		ctatggttcggggagttattataa	ARDRETYGSGSYYKDXRDHYGMDV
EF542585.1	IGHV3-23*01	tgtgcgaa		gaagatcat		gagtggt	AKKIMSGPLDY
GU552746.1	IGHV3-23*01	tgtgcgaaaga	t	gaagagtt		ctacggtgactac	AKDEEFYGDYPTNFDY
Z37302.1	IGHV3-23*04	tgtgcgaaa		ccggggcagc	c	gtatttctatggtt	AKPGQPYFYGCPSEH
EF542605.1	IGHV3-33*05	tgtgcgaaaga	t	attgagcgcatggattgggtgattacccaga		attacttttactac	AKDIERMDWVITQNYFYYGLDV
Z37326.1	IGHV3-23*04	tgtgcgaaa		agcccagac		gtagtagtaccagctgc	AKSPDVVVPAADY
Z37326.1	IGHV3-23*04	tgtgcgaaa		agcccagac		gtagtagtaccagctgc	AKSPDVVVPAADY
AF129750.1	IGHV5-51*01	tgtgcgagaca	tg	agatcggca		accaggttct	ARHEIGNQVLY
DQ926093.1	IGHV3-30*03	tgtgcga		agaccgccgggcaga		tatggcagcgg	AKTAGQIWQRDFYYGMDV
DQ926093.1	IGHV3-30*03	tgtgcga		agaccgccgggcaga		tatggcagcgg	AKTAGQIWQRDFYYGMDV
EF178138.1	IGHV3-23*04	tgtgcgaaaga	t	agcaga		tattacgatatttcgactggttatt	AKDSRYYDISTGYFRGSAFDAFDI
EF178087.1	IGHV4-59*08	tgt		acaagacataccacc		gtggtattgactgctat	TRHTTVVLTAIQFDS
EF178087.1	IGHV4-59*08	tgt		acaagacataccacc		gtggtattgactgctat	TRHTTVVLTAIQFDS
EF542635.1	IGHV4-39*01	tgtgcgagaca	t	tacgg		ttactttggttcggggagttat	ARHYGYFGSGSYPDY
DQ926113.1	IGHV3-23*04	tgtgcgaaag		ggcag		gcagcatct	AKGQAASPYYYMDV
EF542631.1	IGHV4-4*07	tgtgcgag		gcagac		gtatagtagtggctggtac	ARQTYSSGWYDLDY
EF542631.1	IGHV4-4*07	tgtgcgag		gcagac		gtatagtagtggctggtac	ARQTYSSGWYDLDY
EF542623.1	IGHV4-59*08	tgtgcgagac		gcagac		cagtggccgg	ARRRPVAGLFDP
EF542623.1	IGHV4-59*08	tgtgcgagac		gcagac		cagtggccgg	ARRRPVAGLFDP

DQ926073.1	IGHV1-2*02	tgtgcgagag		gagatttacaaaggccga	accagctgctgta	ARGDLQRPNQLLYTGWSDS
EF542642.1	IGHV4-59*01	tgtgcgcgag		gagaggataa	ttcgaggcctaattataa	ARGEDNSRPNYKEDWLDV
EF542642.1	IGHV4-59*01	tgtgcgcgag		gagaggataa	ttcgaggcctaattataa	ARGEDNSRPNYKEDWLDV
EF178010.1	IGHV3-30-3*01	tgtgcgaga		gagagg	tactatggttcggggagttattata	ARERYYGSGSYYMTYYYYGMDV
EF177998.1	IGHV4-30-4*01	tgtgccagaga		gagagagaggg	actatggttcggggagt	ARERERDYGSGSRLVDY
EF177998.1	IGHV4-30-4*01	tgtgccagaga		gagagagaggg	actatggttcggggagt	ARERERDYGSGSRLVDY
EF177998.1	IGHV4-30-4*01	tgtgccagaga		gagagagaggg	actatggttcggggagt	ARERERDYGSGSRLVDY
EF177951.1	IGHV3-33*01	tgtgcgagaga		gagagcccc	ctatggacggg	ARERAPYGRETGGMDV
DQ926105.1	IGHV4-59*01	tgtgcgagag		gaaagagggagcgctctgaat	actaccactactac	ARGKRERSEYYHYYYMDV
DQ926105.1	IGHV4-59*01	tgtgcgagag		gaaagagggagcgctctgaat	actaccactactac	ARGKRERSEYYHYYYMDV
DQ926105.1	IGHV4-59*01	tgtgcgagag		gaaagagggagcgctctgaat	actaccactactac	ARGKRERSEYYHYYYMDV
EF542649.1	IGHV4-30-2*01	tgtgtca		cagag	ggatacggctatgg	VTEGYGYGSSNGFDY
EF178130.1	IGHV1-3*01	tgtgcga		cacagag	cggtggtaac	ATQSGGNVY
EF178130.1	IGHV1-3*01	tgtgcga		cacagag	cggtggtaac	ATQSGGNVY
AF231403.1	IGHV3-23*01	tgtgcgaaaga	tc	cacaggcgg	attactatggttcgggg	AKDPQADYYGSGAPPDY
EF542580.1	IGHV3-74*01	tgttcaagag		caagagg	tgatagtagtggtt	SRARGDSSGSINYYYGMDV
EF542580.1	IGHV3-74*01	tgttcaagag		caagagg	tgatagtagtggtt	SRARGDSSGSINYYYGMDV
DQ926101.1	IGHV4-59*03	tgtgcg		agacat	gtggctacggtta	ARHVATVKLDWFDP
Z37319.1	IGHV3-23*01	tgtgcgaaaga	tc	acgga	cagtggctggtac	AKDHGQWLVRGSQTGDY
AY206995.1	IGHV3-30*03	tgtgcgaaaga	tct	aagagaagctacactg	tattgtggtggtgactgctattc	AKDLREATLYCGGDCYSNYFDL
AY206995.1	IGHV3-30*03	tgtgcgaaaga	tct	aagagaagctacactg	tattgtggtggtgactgctattc	AKDLREATLYCGGDCYSNYFDL
DQ926140.1	IGHV6-1*01	tgtgcaaga		aaaagggggatggccg	gcagctggta	ARKRGMAGSWYVEAFDI

**Allergic rhinitis**							
**Accession #**	**V_H Gene**	**V_H**	**P**	**N1[a]**	**P**	**D_H**	**CDR3 (aa)[b]**
AY971118.1	IGHV5-51*01	tgtgcgag		tgccaaaga		tggaacgac	ASAKDGTTWWWFDP
AY640581.1	IGHV4-61*08	tgtgcgagag		ttagaggaca		agtcggagct	ARVRGQVGALDY
AY640581.1	IGHV4-61*08	tgtgcgagag		ttagaggaca		agtcggagct	ARVRGQVGALDY
AY971105.1	IGHV3-23*05	tgtgcga		ggcggcactc		ttattacgagagtagtcgt	ARRHSYYESSRPGAPIYYYYTMDV
AY640539.1	IGHV3-30-3*01	tgtgcgaga		gatcacggcaaacct	ct	aggatattgtagtagtaccagctgctatac	ARDHGKPLGYCSSTSCYTVRYYYYYGMDV
AY640539.1	IGHV3-30-3*01	tgtgcgaga		gatcacggcaaacct	ct	aggatattgtagtagtaccagctgctatac	ARDHGKPLGYCSSTSCYTVRYYYYYGMDV
AY971116.1	IGHV4-30-4*01	tgtgccaga		ctgagatctcgcttttacgattc		ttggcgcagctat	ARLRSRFYDSWRSYSGSTWHFDL
AY640555.1	IGHV5-a*03	tgtgcgaga		catcttcccacagacac		cagtggctgg	ARHLPTDTSGWLGRGGFDI
AY640555.1	IGHV5-a*03	tgtgcgaga		catcttcccacagacac		cagtggctgg	ARHLPTDTSGWLGRGGFDI

AY640568.1	IGHV3-30*01	tgtgcgaga	caaagaggagtagc	tacctcctactttg	ARQRGVATSYFDN
AY640568.1	IGHV3-30*01	tgtgcgaga	caaagaggagtagc	tacctcctactttg	ARQRGVATSYFDN
AY640568.1	IGHV3-30*01	tgtgcgaga	caaagaggagtagc	tacctcctactttg	ARQRGVATSYFDN
AY971092.1	IGHV1-2*02	tgtgcgagaga	aatcgtgggaaaag	gtgggcgccac	AREIVGKGGRHSFDV
AY640575.1	IGHV5-51*01	tgt	acaagacag	aggagtggct	TRQRSGSPDY
AY640575.1	IGHV5-51*01	tgt	acaagacag	aggagtggct	TRQRSGSPDY
AY971096.1	IGHV3-30*18	tgtgc	aagaggg	ggttcggggagttat	ARGGSGSYLNAPDY
AY971096.1	IGHV3-30*18	tgtgc	aagaggg	ggttcggggagttat	ARGGSGSYLNAPDY
AY971136.1	IGHV1-8*01	tgcgcg	cgagaggc	tgcccagggtgactac	AREAAQGDYALGY
AY640570.1	IGHV3-33*01	tgtgcgagag	cgaga	tatgccgcctcgtcc	ARARYAASSYWFDP
AY971101.1	IGHV3-30-3*01	tgtgcga	aacagt	tggtggggt	AKQLVGSSFGMDV
AY971130.1	IGHV3-30*03	tgtgcga	aacagggca	actatgatagtagtggtcattcct	AKQGNYDSSGHSSSYFDD

**Rheumatoid arthritis**							
**Accession #**	**V_H Gene**	**V_H**	**P**	**N1[a]**	**P**	**D_H**	**CDR3 (aa)[b]**
AY392986.1	IGHV6-1*02	tgtgcaagag		tttccatctatcgacgagactt		atacagtaccaat	ARVSIYRRDLYSTNWWWEWFDP
AY393014.1	IGHV1-18*01	tgtgcgagag		tttctaga		tgtagtactagcagctgcta	ARVSRCSTSSCYKMDY
U85230.1	IGHV3-11*04	tgtgcgagag		ttgggtacggtgacccatttacaggacgtaactccgcg		gactaccttgact	ARVGYGDPFTGRNSADYLDF
AY393467.1	IGHV5-51*01	tgtgcgagac		ttcaaggatattgcac		tggcggtagttgtta	ARLQGYCTGGSCYAGAMDV
L29155.1	IGHV3-21*01	tgtgcgagaga	t	ttaagagg		gcagtggctggtac	ARDLRGQWLVQGEDY
L29155.1	IGHV3-21*01	tgtgcgagaga	t	ttaagagg		gcagtggctggtac	ARDLRGQWLVQGEDY
AY392947.1	IGHV4-39*07	tgtgcgag		ttcggcaggac		acggtgactac	ASSAGHGDYSFLDY
AY392886.1	IGHV2-5*01	tgtg		ttcacggtatagggtcctt		ctatggtggaaactc	VHGIGSFYGGNSEWYFHH
AY393086.1	IGHV5-51*03	tgtgcga		tgtggagaacaatgggtcctctac		atactactggct	AMWRTMGPLHTTGSPLDF
AY393681.1	IGHV1-18*01	tgtgcgagag		tggaacggagac		actatgatcgcagtggtgatt	ARVERRHYDRSGDFLIQCFHY
AY393681.1	IGHV1-18*01	tgtgcgagag		tggaacggagac		actatgatcgcagtggtgatt	ARVERRHYDRSGDFLIQCFHY
AY392847.1	IGHV1-24*01	tgtg		tgggagatcccc		agtggggcta	VGDPQWGYGTRSFDY
AY393284.1	IGHV2-70*04	tgtg		tgcggcatcactatt		ctggttcgac	VRHHYSGSTTSYNYFDH
AY393220.1	IGHV1-2*03	tgtg		tgcgagatatca		ataatgtggtggtgacagc	VRDINNVVVTALGY
AY393459.1	IGHV5-51*01	tgtgcgagac		tgcaaca		atattgcactggtggtagctgctac	ARLQQYCTGGSCYAGAMDV
AY393039.1	IGHV2-26*01	tgtg		tctgcacaaga		ggtgacttccgtccc	VCTRGDFRPLHN
AY393140.1	IGHV1-2*02	tgtgcg		tccctaaagac		ctcggcc	ASLKTSALPFDY
AY393487.1	IGHV6-1*01	tgtgcaaga		tccccaggcagcctga		gtatagcagcagctg	ARSPGSLSIAAAAFFDY
AY393287.1	IGHV2-5*10	tgt		tcccacagcggtctgtcc		tatggtgggggc	SHSGLSYGGGTVAEYFQH

AY393483.1	IGHV6-1*01	tgtg		tacgagatc	tagcggctcgtcc	VRDLAARPNYYYGMDV
AY393475.1	IGHV6-1*01	tgtg		ttagagatctccgtaaggg	agtgggagccac	VRDLRKGVGATGLFDS
AY393475.1	IGHV6-1*01	tgtg		ttagagatctccgtaaggg	agtgggagccac	VRDLRKGVGATGLFDS
AY393376.1	IGHV1-69*01	tgtgcgagaga	tct	tcgaaagcg	cggggagttgttat	ARDLRKRGELLFDYHYGMDV
AY393424.1	IGHV2-5*10	tgtgca		tacagaccgacgggtttttta	tacaatggtccgggcactt	AYRPTGFLYNGPGTLNWFDP
AY393602.1	IGHV1-3*01	tgtgcgagag		gttctgaacgcctctacggctt	gttttcccgcag	ARGSERLYGLFSRSHSTFDY
AY393193.1	IGHV5-51*03	tgtgcgag		gtggagatcg	gcagcagccggta	ARWRSAAAGSWGWFDP
AY392930.1	IGHV3-11*04	tgtgcgag		gtacccgcagaactt	atatgtctact	ARYPQNLYVYSSHYFDY
L19292.1	IGHV1-69*06	tgtgcgcga		gggtcgggggaacacacaa	atatggtggtg	ARGSGEHTNMVVPFDY
L19292.1	IGHV1-69*06	tgtgcgcga		gggtcgggggaacacacaa	atatggtggtg	ARGSGEHTNMVVPFDY
AK301403.1	IGHV3-11*01	tgtgcgag		gggctccgctagg	gtggctacgatt	ARGSARVATIRRSHGSFRFTIDV
AY393533.1	IGHV1-2*02	tgtgcgaaag		gggcgggatactaccttcgct	ctatgattcagggagttat	AKGAGYYLRSMIQGVMGY
AY393259.1	IGHV1-2*02	tgtgcgag		gggcgagattt	actacagtaact	ARGEIYYSNSVYFEY
AY393103.1	IGHV6-1*01	tgttcaag		ggcgggccggata	atagcagtggctgg	SRAGRIIAVAGSDY
AY393137.1	IGHV1-69*01	tgtgcgag		ggcaattcttcctcttgctcggcag	ccggaccc	ARAILPLARQPDPRFDY
AY393657.1	IGHV1-69*01	tgtgcgagag		ggattcgagaaattactttagacaa	ttattactac	ARGIREITLDNYYYHAMDV
AY393657.1	IGHV1-69*01	tgtgcgagag		ggattcgagaaattactttagacaa	ttattactac	ARGIREITLDNYYYHAMDV
AY393572.1	IGHV5-51*01	tgtgcgagac		gggggcagcagct	ggtatagtgggaacta	ARRGSSWYSGNYFSSYYGMDV
AY392998.1	IGHV6-1*01	tgtgcaag		gggggata	tatgatagtagtggtta	ARGIYDSSGYRLDY
AY392990.1	IGHV6-1*01	tgtgcaag		gggggata	tatgatagtagtgatta	ARGIYDSSDYRLDY
AY393101.1	IGHV6-1*01	tgtgcaag		gggggata	tatgatagtagtggtta	ARGIYDSSGYRLDY
AY393208.1	IGHV6-1*01	tgtgcaag		gggggata	tatgatagtagaggtta	ARGIYDSRGYRLDY
AY392992.1	IGHV6-1*01	tgtgcaag		gggggata	tatgatagtagtggtta	ARGIYDSSGYRLDC
Z34960.1	IGHV1-69*01	tgtgcgagaga		gggaaaggcgggg	gactacagtaac	AREGKAGDYSNPFDY
Z34962.1	IGHV1-69*01	tgtgcgagaga		gggaaaggcgggg	gactatagtaac	AREGKAGDYSNPFDY
Z34958.1	IGHV1-69*01	tgtgcgagaga		gggaaaggcgggg	gactacagtaac	AREGKAGDYSNPFDY
Z34906.1	IGHV1-69*01	tgtgcgagaga		gggaaaggcgggg	gactacagtaac	AREGKAGDYSNPFDY
Z34907.1	IGHV1-69*01	tgtgcgagaga		gggaaaggcgggg	gactacaataac	AREGKAGDYNNPFDY
Z34908.1	IGHV1-69*01	tgtgcgagaga		gggaaaggcgggg	gactacagtaa	AREGKAGDYSNPFDY
AY393488.1	IGHV6-1*01	tgtgcaag		gggaaagcctc	tagcagcagctgg	ARGKPLAAAGRPLSPLLDY
AY393476.1	IGHV6-1*01	tgtgcaag		gggaaagcctc	tagcagcagctgg	ARGKPLAAAGRPLSPLFDY
AY393573.1	IGHV5-51*01	tgtgcgag		gcttgaaatacaa	tactctggatcggggagt	ARLEIQYSGSGSRSFDY
AY393606.1	IGHV1-3*01	tgtgcgggag		gcgtcggattc	tttgggggatcggtcg	AGGVGFFGGSVGSFDH
AY393534.1	IGHV1-8*01	tgtgcgagagg		gcctgtaaaacag	atagcagctc	ARGPVKQIAAPDY
AY393470.1	IGHV5-51*01	tgtgcgagac		gcccggat	tttcaggagt	ARRPDFQESLNYFDY
AY393335.1	IGHV6-1*01	tgtgccagaga		gccggat	gagacggc	AREPDETAPPNYDQ

AY393079.1	IGHV4-b*01	tgtgcgaga	gatctagg		tagtgcctacgat	ARDLGSAYDVRKGVKVFYFDY
AY393000.1	IGHV1-69*05	tgtgcgaga	gatcagagt		gtggtagcaccta	ARDQSVVAPSTGAFDI
AY392849.1	IGHV1-3*01	tgtgcgag	gagtgagatcagctcc		tacccggaa	ARSEISSYPEIDF
AY393012.1	IGHV1-69*05	tgtgcgaga	gagcactacagaga		tataacagcagatgg	AREHYRDITADGPDFDY
AY393012.1	IGHV1-69*05	tgtgcgaga	gagcactacagaga		tataacagcagatgg	AREHYRDITADGPDFDY
AY392978.1	IGHV5-51*03	tgtgcgaga	gagaatgaaaa		tgtgatggggg	ARENENVMGGAFDI
AY392978.1	IGHV5-51*03	tgtgcgaga	gagaatgaaaa		tgtgatggggg	ARENENVMGGAFDI
AY393682.1	IGHV1-69*04	tgtgcgaga	gactgcttagaggtccctgtaagtgg		agtattctcagatgc	ARDCLEVPVSGVFSDARENWFDP
AY393682.1	IGHV1-69*04	tgtgcgaga	gactgcttagaggtccctgtaagtgg		agtattctcagatgc	ARDCLEVPVSGVFSDARENWFDP
AY393165.1	IGHV4-59*07	tgtgcgaga	gacctgaagatggccga		acgtaggggg	ARDLKMAERRGSYYYYYMDV
AY393538.1	IGHV1-69*06	tgtgcgaga	gacccagacc		atagtgggagctac	ARDPDHSGSYPGGDY
AY393538.1	IGHV1-69*06	tgtgcgaga	gacccagacc		atagtgggagctac	ARDPDHSGSYPGGDY
AY393052.1	IGHV2-70*11	tgtgcacggat	gaatggcagtgtgaaaggc		agtgcttcttact	ARMNGSVKGSASYSPFDY
AY393052.1	IGHV2-70*11	tgtgcacggat	gaatggcagtgtgaaaggc		agtgcttcttact	ARMNGSVKGSASYSPFDY
AY392860.1	IGHV1-3*01	tgtgcgagag	ggagatttc		attgtagcgtcgactgct	ARGRFHCSVDCFGEVFYAMDV
AY393018.1	IGHV1-46*01	tgtgcga	ctcacggcaatc		tatcagcagctg	ATHGNLSAAALDY
AY393018.1	IGHV1-46*01	tgtgcga	ctcacggcaatc		tatcagcagctg	ATHGNLSAAALDY
AY392912.1	IGHV3-7*01	tg	ctctagacag		gggccaatgatg	SRQGPMMDDSFDI
AY392912.1	IGHV3-7*01	tg	ctctagacag		gggccaatgatg	SRQGPMMDDSFDI
AY392960.1	IGHV5-51*03	tgtgc	ctcgagacgg		tgtgggggcggcggct	ASRRCGGGGSYSH
AY392878.1	IGHV1-69*12	tgtgcgaga	ctcgagaccccaggcgcc		tactactac	ARLETPGAYYYAMDI
AY392878.1	IGHV1-69*12	tgtgcgaga	ctcgagaccccaggcgcc		tactactac	ARLETPGAYYYAMDI
AY393175.1	IGHV4-39*07	tgtgcgaga	cgtcgaaggagagctccgg		gggacagaggg	ARRRRRAPGDRGNWFDP
AY392945.1	IGHV4-61*02	tg	cgtgagaggccccgg		ggatcatttcctggt	VRGPRGSFPGPAWFGP
AY393418.1	IGHV2-5*01	tg	cgtacacagttt		ctattctaactac	VHSFYSNYPRTDYYYGMDV
AY393423.1	IGHV2-5*01	tg	cgtacacagtct		ctattctaactac	VHSLYSNYPRTDYYYGMDV
AY392944.1	IGHV4-4*07	tgtgcgagaga	cggtatagagga	ac	gtggagacaagtttgg	ARDGIEERGDKFGFFDQ
AY392944.1	IGHV4-4*07	tgtgcgagaga	cggtatagagga	ac	gtggagacaagtttgg	ARDGIEERGDKFGFFDQ
AY392946.1	IGHV4-4*07	tgtgcgcgaga	cggtatagagga	ac	gtggagacagttttgg	ARDGIEERGDSFGFFDY
AY392946.1	IGHV4-4*07	tgtgcgcgaga	cggtatagagga	ac	gtggagacagttttgg	ARDGIEERGDSFGFFDY
AJ007328.1	IGHV1-69*05	tgcgc	cggagcgcaacaact		tggtccttat	AGAQQLGPYRFYYGVDV
AY393549.1	IGHV2-26*01	tgtgcacggatac	cggagagtgggcc		tactagtttt	ARIPESGPTSFHYYYYGMDV
AY393565.1	IGHV2-5*01	tgtgcacacag	cgccagag		ggggtctattac	AHSARGGLLHTPFDY
AY393555.1	IGHV2-5*01	tgtgtacacag	cgccagag		ggggtctattac	VHSARGGLLHTPFDY
AY393106.1	IGHV1-69*01	tgtgcgaga	ccgagccacaccaaaagcc		gtagcaggac	ARPSHTKSRSRTTLDY
AY393106.1	IGHV1-69*01	tgtgcgaga	ccgagccacaccaaaagcc		gtagcaggac	ARPSHTKSRSRTTLDY

AY392926.1	IGHV3-64*01	tgtgcgaga		ccctcgaaatccttgatctttcccttgg	gcagtggctggt	ARPSKSLIFPLGSGWFSY
AY393021.1	IGHV1-46*01	tgtgcgagaga	tc	ccccagatgggttc	ggtgactacgcctcc	ARDPPDGFGDYASLPFDY
AY393085.1	IGHV5-51*03	tgtgcgaga		catcacgaggaaagtggc	tgggggtcttttg	ARHHEESGWGSFDY
AY393279.1	IGHV2-5*01	tg		cacacacag	gataagagggg	THRIRGAFDY
AY393279.1	IGHV2-5*01	tg		cacacacag	gataagagggg	THRIRGAFDY
AY393472.1	IGHV5-a*01	tgtgcgag		ccaagaag	atgacaaactttt	ASQEDDKLFGMDL
AY393654.1	IGHV1-69*13	tgtgcgagaga	t	agtagtgcctatagag	gttggaactactac	ARDSSAYRGWNYYYAMDV
AY393283.1	IGHV2-5*10	tgtgcac		accttaagaccacct	cgtccgggggcgt	AHLKTTSSGGVGAFDI
AY393396.1	IGHV2-5*10	tgtgcac		acatagggtcggaatacggc	tacaactggttcga	AHIGSEYGYNWFES
AY392900.1	IGHV2-5*10	tgtgcac		acagcagtcgggcagcaaat	ggtccaacggga	AHSSRAANGPTGYYFDY
AY393306.1	IGHV2-5*04	tgtgcac		acagcaaggcgtggtctattcaaggcaggcagcccggggggg	gtgaatacatctttg	AHSKAWSIQGRQPGGGEYIFDQ
AY393306.1	IGHV2-5*04	tgtgcac		acagcaaggcgtggtctattcaaggcaggcagcccggggggg	gtgaatacatctttg	AHSKAWSIQGRQPGGGEYIFDQ
AY393036.1	IGHV2-5*10	tgtgtac		acaataggggggatac	ttttggagtgg	VHNRGDTFGVALNFDS
AY393055.1	IGHV3-53*02	tgtgtga		aacccacggatgat	gatggccac	VKPTDDDGHRAEYFQY
AY393612.1	IGHV1-69*01	tgtgcgaggga	t	aaagaggttgtggacc	actacggtgacgac	ARDKEVVDHYGDDIAYNYGMDV
AY393612.1	IGHV1-69*01	tgtgcgaggga	t	aaagaggttgtggacc	actacggtgacgac	ARDKEVVDHYGDDIAYNYGMDV
AY393612.1	IGHV1-69*01	tgtgcgaggga	t	aaagaggttgtggacc	actacggtgacgac	ARDKEVVDHYGDDIAYNYGMDV
AY393023.1	IGHV2-5*10	tgttcacgg		atgaaag	gtagtggttattactac	SRMKGSGYYYPFDF
AY393144.1	IGHV2-5*10	tgtgcac		acgagaaaaggg	attgtagtggtggtagctgctactc	AHEKRDCSGGSCYSSYFDY
AY393144.1	IGHV2-5*10	tgtgcac		acgagaaaaggg	attgtagtggtggtagctgctactc	AHEKRDCSGGSCYSSYFDY
AY393266.1	IGHV1-69*06	tgtgcga		tagagaggggt	gatattgttgtggtgggag	AIERGDIVVVGDSAMGTAPDI
AY393266.1	IGHV1-69*06	tgtgcga		tagagaggggt	gatattgttgtggtgggag	AIERGDIVVVGDSAMGTAPDI
AY393269.1	IGHV1-69*06	tgtgcgag		ggcagttga	tatggttcggggagttat	ARAVDMVRGVIFGAYYYYGLDV
U86668.1	IGHV4-4*02	tgtgcgag		gcagagtg	ggatcgggg	ARQSGIGANFDI
U86668.1	IGHV4-4*02	tgtgcgag		gcagagtg	ggatcgggg	ARQSGIGANFDI
AY393670.1	IGHV1-69*04	tgtgcg		gcagaggagta	tagcactacctactatg	AAEEYSTTYYGAGSLES
AY393670.1	IGHV1-69*04	tgtgcg		gcagaggagta	tagcactacctactatg	AAEEYSTTYYGAGSLES
AY393670.1	IGHV1-69*04	tgtgcg		gcagaggagta	tagcactacctactatg	AAEEYSTTYYGAGSLES
AY393032.1	IGHV2-70*11	tgtgcgcggat		gcagacgagg	agcagtggctggtac	ARMQTRSSGWYGSFDI
AY393032.1	IGHV2-70*11	tgtgcgcggat		gcagacgagg	agcagtggctggtac	ARMQTRSSGWYGSFDI
AY393650.1	IGHV1-46*01	tgtgcg		gcaga	tatctcagtgcctggtac	AADISVPGTPYYHYFGMDV
AY393092.1	IGHV5-51*03	tgtgcgaga		gagatgct	ctatggttcgggggttatta	AREMLYGSGGYYPPDAFEV
AY393089.1	IGHV5-51*03	tgtgcgaga		gagatgct	ctatggttcgggggttatta	AREMLYGSGGYYPPDAFEL
AY392980.1	IGHV5-51*03	tgtgcgaga		gagatgct	ctatggttcgggggttatta	AREMLYGSGGYYPPDAFEL
AY393009.1	IGHV1-f*01	tgtgc		gagatccgtc	cgtgtggg	ARSVRVGLYGMDV
AY393642.1	IGHV1-24*01	tgtgc		gagagacaatc	acggttggggt	ARDNHGWGLDI

AY393642.1	IGHV1-24*01	tgtgc		gagagacaatc		acggttggggt	ARDNHGWGLDI
AY393381.1	IGHV1-f*01	tgtgc		gagagtcctc		tattgtggtggtgactgc	ARVLYCGGDCHYFDH
AY393121.1	IGHV1-f*01	tgtgc		gagag		gaggtgattacattt	ARGGDYIWQDY
AY393153.1	IGHV3-11*04	tgtgcgagag		gagaac		tacagctatggtta	ARGELQLWLGNFDY
AY393352.1	IGHV1-69*04	tgtgcga		cagagccccccgttcggtt		tggtgcagggacttttt	ATEPPVRFGAGTFFFD
L29154.1	IGHV3-15*01	tgtacc		cacagaaggaccag		agtagtaccagctgcta	THRRTRVVPAATEAWNVVDY
AY393087.1	IGHV5-51*04	tgtgcgaga		caaaatccccccgagtatagtggc		gcatatcatgatgg	ARQNPPEYSGAYHDGWFDP
AY393088.1	IGHV5-51*03	tgtgcgaga		caaaacccccccgagtatagtggc		gcatatcatgatgg	ARQNPPEYSGAYHDGWFDP
AY393591.1	IGHV6-1*01	tgtgcaaga		caaaa		agcagtggctggtac	ARQKAVAGTHYYYYGMDV
AY393577.1	IGHV6-1*01	tgtgcaaga		caaaa		agcagtggctggtac	ARQKAVAGTHYHYYGMDV
AY393038.1	IGHV2-5*10	tgtgcac		acagaccgg		attactatgatagtattgg	AHRPDYYDSIGALFDY
AY393391.1	IGHV2-5*10	tgtgcac		acagactg		gtgggaactact	AHRLVGTTYFDY
AY392899.1	IGHV2-5*04	tgtgcac		acagacgtgtt		ggggttagaatgac	AHRRVGVRMTENDGFEN
AY393304.1	IGHV2-5*10	tgtgcac		acagacgggtgggg		gactacggtga	AHRRVGDYGDFVTAMDV
AY393386.1	IGHV2-5*10	tgtgcac		acagacgacg		ggatacacctatgg	AHRRRDTPMAFDY
AY392884.1	IGHV2-5*10	tgtgcac		acagacatcgccgtt		cgatttttgggatagt	AHRHRRSIFGIVVPSSAESFDI
AY392884.1	IGHV2-5*10	tgtgcac		acagacatcgccgtt		cgatttttgggatagt	AHRHRRSIFGIVVPSSAESFDI
AY393382.1	IGHV2-5*04	tgtgcac		acagacaag		gcagctcg	AHRQGSSPSYGLDV
AY393382.1	IGHV2-5*04	tgtgcac		acagacaag		gcagctcg	AHRQGSSPSYGLDV
AY393563.1	IGHV2-5*10	tgcgcac		acagagtttcatt		tagcagtgactgg	AHRVSFSSDWNAGYFDY
AY393563.1	IGHV2-5*10	tgcgcac		acagagtttcatt		tagcagtgactgg	AHRVSFSSDWNAGYFDY
AY393660.1	IGHV1-69*06	tgtgcga		aagagcg		gggggagtggagccta	AKERGEWSLRDVKQYVMDV
AY393124.1	IGHV1-46*01	tgtgc		aacagatcatcggg		gcagtggctggtac	ATDHRGSGWYGYYYGMDV
AY393468.1	IGHV5-51*01	tgtgcgagaca	t	aaaagggg		gactacgattactac	ARHKRGTTITTGYFEY

Rheumatoid arthritis synovial

Accession #	V_H Gene	V_H	P	N1[a]	P	D_H	CDR3 (aa)[b]
AY392986.1	IGHV6-1*02	tgtgcaagag		tttccatctatcgacgagactt		atacagtaccaat	ARVSIYRRDLYSTNWWWEWFDP
AY393014.1	IGHV1-18*01	tgtgcgagag		tttctaga		tgtagtactagcagctgcta	ARVSRCSTSSCYKMDY
AY393467.1	IGHV5-51*01	tgtgcgagac		ttcaaggatattgcac		tggcggtagttgtta	ARLQGYCTGGSCYAGAMDV
AY392947.1	IGHV4-39*07	tgtgcgag		ttcggcaggac		acggtgactac	ASSAGHGDYSFLDY
AY392886.1	IGHV2-5*01	tgtg		ttcacggtatagggtcctt		ctatggtggaaactc	VHGIGSFYGGNSEWYFHH
AY393086.1	IGHV5-51*03	tgtgcga		tgtggagaacaatgggtcctctac		atactactggct	AMWRTMGPLHTTGSPLDF

AY392847.1	IGHV1-24*01	tgtg		tgggagatcccc	agtggggcta	VGDPQWGYGTRSFDY
AY393284.1	IGHV2-70*04	tgtg		tgcggcatcactatt	ctggttcgac	VRHHYSGSTTSYNYFDH
AY393220.1	IGHV1-2*03	tgtg		tgcgagatatca	ataatgtggtggtgacagc	VRDINNVVVTALGY
AY393459.1	IGHV5-51*01	tgtgcgagac		tgcaaca	atattgcactggtggtagctgctac	ARLQQYCTGGSCYAGAMDV
AY393039.1	IGHV2-26*01	tgtg		tctgcacaaga	ggtgacttccgtccc	VCTRGDFRPLHN
AY393140.1	IGHV1-2*02	tgtgcg		tccctaaagac	ctcggcc	ASLKTSALPFDY
AY393487.1	IGHV6-1*01	tgtgcaaga		tccccaggcagcctga	gtatagcagcagctg	ARSPGSLSIAAAAFFDY
AY393287.1	IGHV2-5*10	tgt		tcccacagcggtctgtcc	tatggtgggggc	SHSGLSYGGGTVAEYFQH
AY393483.1	IGHV6-1*01	tgtg		tacgagatc	tagcggctcgtcc	VRDLAARPNYYYGMDV
AY393475.1	IGHV6-1*01	tgtg		ttagagatctccgtaaggg	agtgggagccac	VRDLRKGVGATGLFDS
AY393475.1	IGHV6-1*01	tgtg		ttagagatctccgtaaggg	agtgggagccac	VRDLRKGVGATGLFDS
AY393376.1	IGHV1-69*01	tgtgcgagaga	tct	tcgaaagcg	cggggagttgttat	ARDLRKRGELLFDYHYGMDV
AY393424.1	IGHV2-5*10	tgtgca		tacagaccgacgggtttttta	tacaatggtccgggcactt	AYRPTGFLYNGPGTLNWFDP
AY393193.1	IGHV5-51*03	tgtgcgag		gtggagatcg	gcagcagccggta	ARWRSAAAGSWGWFDP
AY392930.1	IGHV3-11*04	tgtgcgag		gtacccgcagaactt	atatgtctact	ARYPQNLYVYSSHYFDY
AK301403.1	IGHV3-11*01	tgtgcgag		gggctccgctagg	gtggctacgatt	ARGSARVATIRRSHGSFRFTIDV
AY393533.1	IGHV1-2*02	tgtgcgaaag		gggcgggatactaccttcgct	ctatgattcagggagttat	AKGAGYYLRSMIQGVMGY
AY393259.1	IGHV1-2*02	tgtgcgag		gggcgagattt	actacagtaact	ARGEIYYSNSVYFEY
AY393103.1	IGHV6-1*01	tgttcaag		ggcgggccggata	atagcagtggctgg	SRAGRIIAVAGSDY
AY393137.1	IGHV1-69*01	tgtgcgag		ggcaattcttcctcttgctcggcag	ccggaccc	ARAILPLARQPDPRFDY
AY393572.1	IGHV5-51*01	tgtgcgagac		gggggcagcagct	ggtatagtgggaacta	ARRGSSWYSGNYFSSYYGMDV
AY392998.1	IGHV6-1*01	tgtgcaag		gggggata	tatgatagtagtggtta	ARGIYDSSGYRLDY
AY392990.1	IGHV6-1*01	tgtgcaag		gggggata	tatgatagtagtgatta	ARGIYDSSDYRLDY
AY393101.1	IGHV6-1*01	tgtgcaag		gggggata	tatgatagtagtggtta	ARGIYDSSGYRLDY
AY393208.1	IGHV6-1*01	tgtgcaag		gggggata	tatgatagtagaggtta	ARGIYDSRGYRLDY
AY392992.1	IGHV6-1*01	tgtgcaag		gggggata	tatgatagtagtggtta	ARGIYDSSGYRLDC
AY393488.1	IGHV6-1*01	tgtgcaag		ggggaaagcctc	tagcagcagctgg	ARGKPLAAAGRPLSPLLDY
AY393476.1	IGHV6-1*01	tgtgcaag		ggggaaagcctc	tagcagcagctgg	ARGKPLAAAGRPLSPLFDY
AY393573.1	IGHV5-51*01	tgtgcgag		gcttgaaatacaa	tactctggatcggggagt	ARLEIQYSGSGSRSFDY
AY393534.1	IGHV1-8*01	tgtgcgagagg		gcctgtaaaacag	atagcagctc	ARGPVKQIAAPDY
AY393470.1	IGHV5-51*01	tgtgcgagac		gcccggat	tttcaggagt	ARRPDFQESLNYFDY
AY393335.1	IGHV6-1*01	tgtgccagaga		gccggat	gagacggc	AREPDETAPPNYDQ
AY393079.1	IGHV4-b*01	tgtgcgaga		gatctagg	tagtgcctacgat	ARDLGSAYDVRKGVKVFYFDY
AY393000.1	IGHV1-69*05	tgtgcgaga		gatcagagt	gtggtagcaccta	ARDQSVVAPSTGAFDI
AY392849.1	IGHV1-3*01	tgtgcgag		gagtgagatcagctcc	tacccggaa	ARSEISSYPEIDF
AY393012.1	IGHV1-69*05	tgtgcgaga		gagcactacagaga	tataacagcagatgg	AREHYRDITADGPDFDY

AY393012.1	IGHV1-69*05	tgtgcgaga		gagcactacagaga		tataacagcagatgg	AREHYRDITADGPDFDY
AY392978.1	IGHV5-51*03	tgtgcgaga		gagaatgaaaa		tgtgatggggg	ARENENVMGGAFDI
AY392978.1	IGHV5-51*03	tgtgcgaga		gagaatgaaaa		tgtgatggggg	ARENENVMGGAFDI
AY393165.1	IGHV4-59*07	tgtgcgaga		gacctgaagatggccga		acgtaggggg	ARDLKMAERRGSYYYYYMDV
AY393538.1	IGHV1-69*06	tgtgcgaga		gacccagacc		atagtgggagctac	ARDPDHSGSYPGGDY
AY393538.1	IGHV1-69*06	tgtgcgaga		gacccagacc		atagtgggagctac	ARDPDHSGSYPGGDY
AY393052.1	IGHV2-70*11	tgtgcacggat		gaatggcagtgtgaaaggc		agtgcttcttact	ARMNGSVKGSASYSPFDY
AY393052.1	IGHV2-70*11	tgtgcacggat		gaatggcagtgtgaaaggc		agtgcttcttact	ARMNGSVKGSASYSPFDY
AY392860.1	IGHV1-3*01	tgtgcgagag		ggagatttc		attgtagcgtcgactgct	ARGRFHCSVDCFGEVFYAMDV
AY393018.1	IGHV1-46*01	tgtgcga		ctcacggcaatc		tatcagcagctg	ATHGNLSAAALDY
AY393018.1	IGHV1-46*01	tgtgcga		ctcacggcaatc		tatcagcagctg	ATHGNLSAAALDY
AY392912.1	IGHV3-7*01	tg		ctctagacag		gggccaatgatg	SRQGPMMDDSFDI
AY392912.1	IGHV3-7*01	tg		ctctagacag		gggccaatgatg	SRQGPMMDDSFDI
AY392960.1	IGHV5-51*03	tgtgc		ctcgagacgg		tgtgggggcggcggct	ASRRCGGGGSYSH
AY392878.1	IGHV1-69*12	tgtgcgaga		ctcgagacccaggcgcc		tactactac	ARLETPGAYYYAMDI
AY392878.1	IGHV1-69*12	tgtgcgaga		ctcgagacccaggcgcc		tactactac	ARLETPGAYYYAMDI
AY393175.1	IGHV4-39*07	tgtgcgaga		cgtcgaaggagagctccgg		gggacagaggg	ARRRRRAPGDRGNWFDP
AY392945.1	IGHV4-61*02	tg		cgtgagagggccccgg		ggatcatttcctggt	VRGPRGSFPGPAWFGP
AY393418.1	IGHV2-5*01	tg		cgtacacagttt		ctattctaactac	VHSFYSNYPRTDYYYGMDV
AY393423.1	IGHV2-5*01	tg		cgtacacagtct		ctattctaactac	VHSLYSNYPRTDYYYGMDV
AY392944.1	IGHV4-4*07	tgtgcgagaga		cggtatagagga	ac	gtggagacaagtttgg	ARDGIEERGDKFGFFDQ
AY392944.1	IGHV4-4*07	tgtgcgagaga		cggtatagagga	ac	gtggagacaagtttgg	ARDGIEERGDKFGFFDQ
AY392946.1	IGHV4-4*07	tgtgcgcgaga		cggtatagagga	ac	gtggagacagttttgg	ARDGIEERGDSFGFFDY
AY392946.1	IGHV4-4*07	tgtgcgcgaga		cggtatagagga	ac	gtggagacagttttgg	ARDGIEERGDSFGFFDY
AY393549.1	IGHV2-26*01	tgtgcacggatac		cggagagtgggcc		tactagtttt	ARIPESGPTSFHYYYYGMDV
AY393565.1	IGHV2-5*01	tgtgcacacag		cgccagag		ggggtctattac	AHSARGGLLHTPFDY
AY393555.1	IGHV2-5*01	tgtgtacacag		cgccagag		ggggtctattac	VHSARGGLLHTPFDY
AY393106.1	IGHV1-69*01	tgtgcgaga		ccgagccacaccaaaagcc		gtagcaggac	ARPSHTKSRSRTTLDY
AY393106.1	IGHV1-69*01	tgtgcgaga		ccgagccacaccaaaagcc		gtagcaggac	ARPSHTKSRSRTTLDY
AY392926.1	IGHV3-64*01	tgtgcgaga		ccctcgaaatccttgatctttcccttgg		gcagtggctggt	ARPSKSLIFPLGSGWFSY
AY393021.1	IGHV1-46*01	tgtgcgagaga	tc	ccccagatgggttc		ggtgactacgcctcc	ARDPPDGFGDYASLPFDY
AY393085.1	IGHV5-51*03	tgtgcgaga		catcacgaggaaagtggc		tgggggtcttttg	ARHHEESGWGSFDY
AY393279.1	IGHV2-5*01	tg		cacacacag		gataagagggg	THRIRGAFDY
AY393279.1	IGHV2-5*01	tg		cacacacag		gataagagggg	THRIRGAFDY
AY393472.1	IGHV5-a*01	tgtgcgag		ccaagaag		atgacaaactttt	ASQEDDKLFGMDL
AY393283.1	IGHV2-5*10	tgtgcac		accttaagaccacct		cgtccgggggcgt	AHLKTTSSGGVGAFDI

AY393396.1	IGHV2-5*10	tgtgcac	acatagggtcggaatacggc	tacaactggttcga	AHIGSEYGYNWFES
AY392900.1	IGHV2-5*10	tgtgcac	acagcagtcgggcagcaaat	ggtccaacggga	AHSSRAANGPTGYYFDY
AY393306.1	IGHV2-5*04	tgtgcac	acagcaaggcgtggtctattcaaggcaggcagcccggggggg	gtgaatacatctttg	AHSKAWSIQGRQPGGGEYIFDQ
AY393306.1	IGHV2-5*04	tgtgcac	acagcaaggcgtggtctattcaaggcaggcagcccggggggg	gtgaatacatctttg	AHSKAWSIQGRQPGGGEYIFDQ
AY393036.1	IGHV2-5*10	tgtgtac	acaataggggggatac	ttttggagtgg	VHNRGDTFGVALNFDS
AY393055.1	IGHV3-53*02	tgtgtga	aacccacggatgat	gatggccac	VKPTDDDGHRAEYFQY
AY393023.1	IGHV2-5*10	tgttcacgg	atgaaag	gtagtggttattactac	SRMKGSGYYYPFDF
AY393144.1	IGHV2-5*10	tgtgcac	acgagaaaaggg	attgtagtggtggtagctgctactc	AHEKRDCSGGSCYSSYFDY
AY393144.1	IGHV2-5*10	tgtgcac	acgagaaaaggg	attgtagtggtggtagctgctactc	AHEKRDCSGGSCYSSYFDY
AY393266.1	IGHV1-69*06	tgtgcga	tagagaggggt	gatattgttgtggtgggag	AIERGDIVVVGDSAMGTAPDI
AY393266.1	IGHV1-69*06	tgtgcga	tagagaggggt	gatattgttgtggtgggag	AIERGDIVVVGDSAMGTAPDI
AY393269.1	IGHV1-69*06	tgtgcgag	ggcagttga	tatggttcggggagttat	ARAVDMVRGVIFGAYYYYGLDV
AY393032.1	IGHV2-70*11	tgtgcgcggat	gcagacgagg	agcagtggctggtac	ARMQTRSSGWYGSFDI
AY393032.1	IGHV2-70*11	tgtgcgcggat	gcagacgagg	agcagtggctggtac	ARMQTRSSGWYGSFDI
AY393092.1	IGHV5-51*03	tgtgcgaga	gagatgct	ctatggttcggggggttatta	AREMLYGSGGYYPPDAFEV
AY393089.1	IGHV5-51*03	tgtgcgaga	gagatgct	ctatggttcggggggttatta	AREMLYGSGGYYPPDAFEL
AY392980.1	IGHV5-51*03	tgtgcgaga	gagatgct	ctatggttcggggggttatta	AREMLYGSGGYYPPDAFEL
AY393009.1	IGHV1-f*01	tgtgc	gagatccgtc	cgtgtggg	ARSVRVGLYGMDV
AY393381.1	IGHV1-f*01	tgtgc	gagagtcctc	tattgtggtggtgactgc	ARVLYCGGDCHYFDH
AY393121.1	IGHV1-f*01	tgtgc	gagag	gaggtgattacattt	ARGGDYIWQDY
AY393153.1	IGHV3-11*04	tgtgcgagag	gagaac	tacagctatggtta	ARGELQLWLGNFDY
AY393352.1	IGHV1-69*04	tgtgcga	cagagccccccgttcggtt	tggtgcagggactttt	ATEPPVRFGAGTFFFD
AY393087.1	IGHV5-51*04	tgtgcgaga	caaaatccccccgagtatagtggc	gcatatcatgatgg	ARQNPPEYSGAYHDGWFDP
AY393088.1	IGHV5-51*03	tgtgcgaga	caaaacccccccgagtatagtggc	gcatatcatgatgg	ARQNPPEYSGAYHDGWFDP
AY393591.1	IGHV6-1*01	tgtgcaaga	caaaa	agcagtggctggtac	ARQKAVAGTHYYYYGMDV
AY393577.1	IGHV6-1*01	tgtgcaaga	caaaa	agcagtggctggtac	ARQKAVAGTHYHYYGMDV
AY393038.1	IGHV2-5*10	tgtgcac	acagaccgg	attactatgatagtattgg	AHRPDYYDSIGALFDY
AY393391.1	IGHV2-5*10	tgtgcac	acagactg	gtgggaactact	AHRLVGTTYFDY
AY392899.1	IGHV2-5*04	tgtgcac	acagacgtgtt	ggggttagaatgac	AHRRVGVRMTENDGFEN
AY393304.1	IGHV2-5*10	tgtgcac	acagacgggtgggg	gactacggtga	AHRRVGDYGDFVTAMDV
AY393386.1	IGHV2-5*10	tgtgcac	acagacgacg	ggatacacctatgg	AHRRRDTPMAFDY
AY392884.1	IGHV2-5*10	tgtgcac	acagacatcgccgtt	cgatttttgggatagt	AHRHRRSIFGIVVPSSAESFDI
AY392884.1	IGHV2-5*10	tgtgcac	acagacatcgccgtt	cgatttttgggatagt	AHRHRRSIFGIVVPSSAESFDI
AY393382.1	IGHV2-5*04	tgtgcac	acagacaag	gcagctcg	AHRQGSSPSYGLDV
AY393382.1	IGHV2-5*04	tgtgcac	acagacaag	gcagctcg	AHRQGSSPSYGLDV
AY393563.1	IGHV2-5*10	tgcgcac	acagagtttcatt	tagcagtgactgg	AHRVSFSSDWNAGYFDY

AY393563.1	IGHV2-5*10	tgcgcac		acagagtttcatt		tagcagtgactgg	AHRVSFSSDWNAGYFDY
AY393124.1	IGHV1-46*01	tgtgc		aacagatcatcggg		gcagtggctggtac	ATDHRGSGWYGYYYGMDV
AY393468.1	IGHV5-51*01	tgtgcgagaca	t	aaaagggg		gactacgattactac	ARHKRGTTITTGYFEY

Systemic lupus erythematosus

Accession #	V_H Gene	V_H	P	N1[a]	P	D_H	CDR3 (aa)[b]
X70488.1	IGHV1-2*02	tgtgcgaga		tctcccctcacgg		tgacccccgtgg	ARSPLTVTPVGYFDS
AY204759.1	IGHV4-34*01	tgtgcgagagg		tcggcac		tggttcagggag	ARGRHWFRELQIRHWFDP
EU666280.1	IGHV5-51*01	tgtgcgagac		tcacagac		gtggatgg	ARLTDVDGH
EU665799.1	IGHV5-51*01	tgtgcgagaca		gcggata		tatgatagtagtggttatt	ARQRIYDSSGYYY
AY204755.1	IGHV4-34*01	tgtgcga		cccacag	ac	gtggatacagctatggttac	ATHRRGYSYGYAIDY
Z49141.1	IGHV4-34*01	tgtgcgagag		tacggcgatcgggtagg		gtagtagtaccagctgc	ARVRRSGRVVVPAAPRNRDAFDI
EU665939.1	IGHV1-69*01	tgtgcgagag		gatac		gggagaccagtggctggcac	ARGYGRPVAGTLTYYYYHMDV
EU665982.1	IGHV1-69*01	tgtgcgggag		gatac		gggagaccagtggctggcac	AGGYGRPVAGTLTYYYYHMDV
AF082198.1	IGHV3-23*01	tgtgcg		cacggggga		aactggaact	AHGGNWNWNFGL
EU666009.1	IGHV2-5*10	tgtgcac		acagaccct		actacggtgacta	AHRPYYGDYVDY
EU666051.1	IGHV2-5*10	tgtgcac		acagaccct		actacggtgacta	AHRPYYGDYVEY
AJ305214	IGHV4-34*01	tgtgcgagagg	c	ttcggcgggagagattactctg		atgctt	ARGFGGRDYSDAXEI
AJ305214	IGHV4-34*01	tgtgcgagagg	c	ttcggcgggagagattactctg		atgctt	ARGFGGRDYSDAXEI
AJ305207	IGHV3-13*01	tgtgcaagag		tcgtccgagatgc		cagtgggtggt	ARVVRDASGWFHYDS
AJ305201	IGHV4-61*08	tgtgcgagag		ggtggtattacgaaac		tttgaccggt	ARGWYYETLTGDGRLEGSGGGLDS
AJ305202	IGHV4-61*07	tgtgcggga		gggtggttctatgaaac		tttgactgg	AGGWFYETLTGSGRLEGSGGGLDS

Diabetes

Accession #	V_H Gene	V_H	P	N1[a]	P	D_H	CDR3 (aa)[b]
D16837.1	IGHV1-69*01	tgtgcgaga		tcgaaagttaattt		cagtggctggtac	ARSKVNFSGWYYY
Z46847.1	IGHV4-59*01	tgtgcgagaga		ggggaaagtatc		cagtggctgg	AREGKVSSGWRLWYFDL
L07312.1	IGHV3-11*01	tgtgcgagaga	tc	gccccggaagagtccgatcgggaagt		ggagctattac	ARDRPGRVRSGSGAITH

Systemic lupus erythematosus plasmablasts

Accession #	V_H Gene	V_H	P	N1[a]	P	D_H	CDR3 (aa)[b]
AJ305214	IGHV4-34*01	tgtgcgagagg	c	ttcggcggGagagattactctg		atgctt	ARGFGGRDYSDAXEI
AJ305214	IGHV4-34*01	tgtgcgagagg	c	ttcggcgggagagattactctg		atgctt	ARGFGGRDYSDAXEI
AJ305207	IGHV3-13*01	tgtgcaagag		tcgtccgagatgc		cagtgggtggt	ARVVRDASGWFHYDS
AJ305201	IGHV4-61*08	tgtgcgagag		ggtggtattacgaaac		tttgaccggt	ARGWYYETLTGDGRLEGSGGGLDS
AJ305202	IGHV4-61*07	tgtgcggga		gggtggttctatgaaac		tttgactgg	AGGWFYETLTGSGRLEGSGGGLDS

Anti-DNA/Histone Ab

Accession #	V_H Gene	V_H	P	N1[a]	P	D_H	CDR3 (aa)[b]
X70488.1	IGHV1-2*02	tgtgcgaga		tctcccctcacgg		tgacccccgtgg	ARSPLTVTPVGYFDS
X70467.1	IGHV3-30*04	tgtgcgag		tccggggaaag		tggagaagtgggagct	ASPGKVEKWELPFDY
AJ401164.1	IGHV3-15*01	tgt		gcaagaaat		tatacgcgga	ARNYTRTLRYSKR
AJ401164.1	IGHV3-15*01	tgt		gcaagaaat		tatacgcgga	ARNYTRTLRYSKR
AJ401163.1	IGHV3-15*01	tgt		gcaaga		aggtatactaa	ARRYTK
AJ306678.1	IGHV3-9*01	tg		cacaaaagggag		ggaggtgataactctt	TKGREVITLNWFHP
AF082198.1	IGHV3-23*01	tgtgcg		cacggggga		aactggaact	AHGGNWNWNFGL
AJ401162.1	IGHV3-23*04	tgtgc		aagatc		tgtgcgggaga	ARSVREKRRRSKAD

Rheumatoid factor

Accession #	V_H Gene	V_H	P	N1[a]	P	D_H	CDR3 (aa)[b]
U85230.1	IGHV3-11*04	tgtgcgagag		ttgggtacggtgacccatttacaggacgtaactccgcg		gactaccttgact	ARVGYGDPFTGRNSADYLDF
L29155.1	IGHV3-21*01	tgtgcgagaga	t	ttaagagg		gcagtggctggtac	ARDLRGQWLVQGEDY
L29155.1	IGHV3-21*01	tgtgcgagaga	t	ttaagagg		gcagtggctggtac	ARDLRGQWLVQGEDY
L19292.1	IGHV1-69*06	tgtgcgcga		gggtcgggggaacacacaa		atatggtggtg	ARGSGEHTNMVVPFDY
L19292.1	IGHV1-69*06	tgtgcgcga		gggtcgggggaacacacaa		atatggtggtg	ARGSGEHTNMVVPFDY
Z34960.1	IGHV1-69*01	tgtgcgagaga		gggaaaggcgggg		gactacagtaac	AREGKAGDYSNPFDY
Z34962.1	IGHV1-69*01	tgtgcgagaga		gggaaaggcgggg		gactatagtaac	AREGKAGDYSNPFDY

Z34958.1	IGHV1-69*01	tgtgcgagaga		gggaaaggcgggg		gactacagtaac	AREGKAGDYSNPFDY
Z34906.1	IGHV1-69*01	tgtgcgagaga		gggaaaggcgggg		gactacagtaac	AREGKAGDYSNPFDY
Z34907.1	IGHV1-69*01	tgtgcgagaga		gggaaaggcgggg		gactacaataac	AREGKAGDYNNPFDY
Z34908.1	IGHV1-69*01	tgtgcgagaga		gggaaaggcgggg		gactacagtaa	AREGKAGDYSNPFDY
U86668.1	IGHV4-4*02	tgtgcgag		gcagagtg		ggatcgggg	ARQSGIGANFDI
U86668.1	IGHV4-4*02	tgtgcgag		gcagagtg		ggatcgggg	ARQSGIGANFDI
L29154.1	IGHV3-15*01	tgtacc		cacagaaggaccag		agtagtaccagctgcta	THRRTRVVPAATEAWNVVDY
**Anti-Rh(D) Ab**							
**Accession #**	**V_H Gene**	**V_H**	**P**	**N1[a]**	**P**	**D_H**	**CDR3 (aa)[b]**
DQ431495.1	IGHV3-48*03	tgtgcgagaga	t	ttgtacgg		tgacttcgatgttaagtcc	ARDLYGDFDVKSYYYYVMDV
DQ431497.1	IGHV3-48*03	tgtgcgagaga	t	ttgtacgg		tgactacgatcctaagtcc	ARDLYGDYDPKSYYYYAMDV
DQ431489.1	IGHV3-48*03	tgtgcgagaga	t	ttgtacgg		tgactacgatcctaagtcc	ARDLYGDYDPKSYYYHAMDV
U43762.1	IGHV1-2*02	tgtgcgagag		ttcgacggccggggcaggtcgccccc		ttgtactactacc	ARVRRPGQVAPLYYYPMDV
DQ431608.1	IGHV3-33*01	tgtgcgaaaga		gcacggctat		tatagcagcagctggta	AKEHGYYSSSWYRNYYYYAMDV
DQ431603.1	IGHV3-33*01	tgtgcgaaaga		gcacggctat		tatagcagcagctggtac	AKEHGYYSSSWYRNYYYYAMDV
DQ431494.1	IGHV3-48*03	tgtgcga		caaatttgtacgg		tgactacgatcctaagtc	ATNLYGDYDPKSFYYYAMGV
A68517.1	IGHV3-30*02	t		attgtgcgagagagaaggcgcttcggggaatcagca		gatacaactat	CAREKALRGISRYNYYLDV
A68517.1	IGHV3-30*02	t		attgtgcgagagagaaggcgcttcggggaatcagca		gatacaactat	CAREKALRGISRYNYYLDV
A68517.1	IGHV3-30*02	t		attgtgcgagagagaaggcgcttcggggaatcagca		gatacaactat	CAREKALRGISRYNYYLDV
U43763.1	IGHV3-30*03	tgtgcgcgaga		aagaactgtaagag		gggggtggtcacgtt	ARERTVRGGWSRFLYYMDV
DQ431622.1	IGHV3-30-3*01	tgtgcgaga		gagagtactcta		tatagcagcagctggtac	ARESTLYSSSWYRRYYYYSMDV
DQ431609.1	IGHV3-21*01	tgtgcgagag		gagagcctctaaac		tatgattacatttggggagggtatcgtt	ARGEPLNYDYIWGGYRFTIH
DQ431611.1	IGHV3-21*01	tgtgcgagag		gagagcctctaaac		tatgattacatttggggaaggtctcgt	ARGEPLNYDYIWGRSRLTIH
DQ431613.1	IGHV3-21*01	tgtgcgagag		gagagcctctaa		attatgattacatttggggaaggtctcgtt	ARGEPLNYDYIWGRSRFTIP
DQ431487.1	IGHV3-48*03	tgtacg		cgagatttgtacgg		tgactacgatcctaagtcc	TRDLYGDYDPKSYYYHAMDV
DQ431487.1	IGHV3-48*03	tgtacg		cgagatttgtacgg		tgactacgatcctaagtcc	TRDLYGDYDPKSYYYHAMDV
U43759.1	IGHV3-33*01	tgtgcgagaga		ccagattgggaagc		ttatcatta	ARDQIGKLIIKYYYYMDV
**Anti-Myelin Ab**							
**Accession #**	**V_H Gene**	**V_H**	**P**	**N1[a]**	**P**	**D_H**	**CDR3 (aa)[b]**
Z18322.1	IGHV3-15*01	tgtaccacaga	tct	aagaacgagagaccctt		tagtgggaact	TTDLRTRDPLVGTA

Z18322.1	IGHV3-15*01	tgtaccacaga	tct	aagaacgagagacccctt		tagtgggaact	TTDLRTRDPLVGTA
Z18322.1	IGHV3-15*01	tgtaccacaga	tct	aagaacgagagacccctt		tagtgggaact	TTDLRTRDPLVGTA
Z18323.1	IGHV3-15*01	tgtaccacaga		gagaaaaagcaaagtct		ctttggttcggggaattattataac	TTERKSKVSLVRGIIITVRERFDI
Z18323.1	IGHV3-15*01	tgtaccacaga		gagaaaaagcaaagtct		ctttggttcggggaattattataac	TTERKSKVSLVRGIIITVRERFDI

Anti-Acetylcholine Ab

Accession #	V_H Gene	V_H	P	N1[a]	P	D_H	CDR3 (aa)[b]
AY033527.1	IGHV3-21*01	tgtg		ttcgagagtgggggagtagattc		atcactacgtttcgggg	VREWGSRFITTFRGLPHFDL
AY033526.1	IGHV3-21*01	tgtg		ttcgagagtggggaagtagattc		atcactacgtttcgggg	VREWGSRFITTFRGLPHFDL
AY033529.1	IGHV3-21*01	tgtg		ttcgagagtggggaagtagattc		atcactacgtttcgggg	VREWGSRFITTFRGLPHFDL
AY033528.1	IGHV3-21*01	tgtg		ttcgagaatggggaagcagattcatc		acaacatttcgggg	VREWGSRFITTFRGLPHFDL
AY033528.1	IGHV3-21*01	tgtg		ttcgagaatggggaagcagattcatc		acaacatttcgggg	VREWGSRFITTFRGLPHFDL
AY033530.1	IGHV3-21*01	tgtg		tgcgagaatggggcagtagattc		atcacttcgtttcgggg	VREWGSRFITSFRGLPHFDL
AY033530.1	IGHV3-21*01	tgtg		tgcgagaatggggcagtagattc		atcacttcgtttcgggg	VREWGSRFITSFRGLPHFDL

Rabies virus

Accession #	V_H Gene	V_H	P	N1[a]	P	D_H	CDR3 (aa)[b]
AY941981.1	IGHV3-30*04	tgtgtga		tacggaccccc		cagttcgccc	VIRTPQFAQYYFDS
AY941981.1	IGHV3-30*04	tgtgtga		tacggaccccc		cagttcgccc	VIRTPQFAQYYFDS
AY941959.1	IGHV4-59*01	tgtgtgagaga		gacggagaaa		tactatgatagaagtggttat	VRETEKYYDRSGYPYYYYYMDV
AY941959.1	IGHV4-59*01	tgtgtgagaga		gacggagaaa		tactatgatagaagtggttat	VRETEKYYDRSGYPYYYYYMDV
AY941893.1	IGHV4-59*01	tgcgcgagaga		gaaggagaaa		tactctgatagaagcggttatt	AREKEKYSDRSGYSYYYYYMDV
AY172958.1	IGHV2-5*04	tgtg		cgcacagacaac		atatcagcagct	AHRQHISSFPWFDS
AY172958.1	IGHV2-5*04	tgtg		cgcacagacaac		atatcagcagct	AHRQHISSFPWFDS
AY941961.1	IGHV3-30*03	tgtgcgaaag		ggcagatgg		aggatttttggagtggtt	AKGQMEDFWSGSH
AY941892.1	IGHV3-30*03	tgtgcgaaag		gagactc		agtgggagcta	AKGDSVGAIHY
AY941939.1	IGHV3-9*01	tgtgc		gagaaacccctgctca		tatgatagtagtggttattact	ARNPCSYDSSGYYCASDAFDI
AY941908.1	IGHV3-30*03	tgtgc		aaaagtc		gctgtggctgg	AKVAVAGESFDS

EBV

Accession #	V_H Gene	V_H	P	N1[a]	P	D_H	CDR3 (aa)[b]
Z46342.1	IGHV4-34*01	tgtgcgagag		tcgccacggg		ttacgatttttggagtggttat	ARVATGYDFWSGYHRLDY
Z46316.1	IGHV4-34*01	tgtgcgagagg		ggattctatggagactgcgccgtcc		tacgatttttggagtggttattat	ARGDSMETAPSYDFWSGYYLDAFDI
AJ506409.1	IGHV3-23*01	tgtg		gcagagagttgggg		agcaatggttggtac	GRELGSNGWYTLDS
AJ506409.1	IGHV3-23*01	tgtg		gcagagagttgggg		agcaatggttggtac	GRELGSNGWYTLDS
AJ506409.1	IGHV3-23*01	tgtg		gcagagagttgggg		agcaatggttggtac	GRELGSNGWYTLDS

HIV

Accession #	V_H Gene	V_H	P	N1[a]	P	D_H	CDR3 (aa)[b]
L08903.1	IGHV1-3*01	tgtgcgagag		tggg agag		tggacttgggatg	ARVGEWTWDDSPQDNYYMDV
L08902.1	IGHV1-3*01	tgtgcgagag		tggg agag		tggacttgggatg	ARVGEWTWDDSPQDNYYMDV
L09077.1	IGHV1-3*01	tgtgcgagag		tggg agag		tggacttgggatg	ARVGEWTWDDFPQDNYYMDV
AF471288.1	IGHV3-23*04	tgtgtgaa		tgataaagagagagatgacg		gtggctgg	VNDKERDDGGWRDP
AF471288.1	IGHV3-23*04	tgtgtgaa		tgataaagagagagatgacg		gtggctgg	VNDKERDDGGWRDP
AF471288.1	IGHV3-23*04	tgtgtgaa		tgataaagagagagatgacg		gtggctgg	VNDKERDDGGWRDP
AF471288.1	IGHV3-23*04	tgtgtgaa		tgataaagagagagatgacg		gtggctgg	VNDKERDDGGWRDP
AF471288.1	IGHV3-23*04	tgtgtgaa		tgataaagagagagatgacg		gtggctgg	VNDKERDDGGWRDP
AF471520.1	IGHV3-64*01	tgtg		tggaaagtatgg		ctttgcttcaggga	VESMALLQGPVGY
AM947637.1	IGHV4-30-2*05	cgtgccagag		tcttctctgaaatctttgc		ttacgatttttggagtggttatt	ARVFSEIFAYDFWSGYCYYFDY
AF471339.1	IGHV3-23*04	tgtgcgagaga	t	tcggtcagag		tgactggggagg	ARDSVRVTGEGDFNY
AM947589.1	IGHV3-13*01	tgtg		ttagagcttccggccgttgtg		ctgatggtctatgttattcc	VRASGRCADGLCYSYYYNGMDV
AF471452.1	IGHV3-33*01	tgt		tccagagatcgtc		acggtgactac	SRDRHGDYRFDI
AF471452.1	IGHV3-33*01	tgt		tccagagatcgtc		acggtgactac	SRDRHGDYRFDI
AF471492.1	IGHV3-48*03	tgtg		tcagaga		tggattcggtgtct	VRDGFGVYDFDY
AF471539.1	IGHV3-66*01	tgtg		tcagacatg		ttgcgtttcagggagt	VRHVAFQGVARDY
AF471539.1	IGHV3-66*01	tgtg		tcagacatg		ttgcgtttcagggagt	VRHVAFQGVARDY
AF471182.1	IGHV3-7*01	tgtgcgagag		tacggattg		acgattattggagtggt	ARVRIDDYWSGDAYGMDV
AF471182.1	IGHV3-7*01	tgtgcgagag		tacggattg		acgattattggagtggt	ARVRIDDYWSGDAYGMDV
EU794436.1	IGHV1-18*01	tgtgtgagag		taagagata	a	tgaacagggagactac	VRVRDNEQGDYGQLDL
EU794436.1	IGHV1-18*01	tgtgtgagag		taagagata	a	tgaacagggagactac	VRVRDNEQGDYGQLDL
EU794433.1	IGHV3-7*02	tgtgcgag		gtggatcggggaaccccaaaaagaagga		tactactatgat	ARWIGEPQKEGYYYDYAMDV
EU794433.1	IGHV3-7*02	tgtgcgag		gtggatcggggaaccccaaaaagaagga		tactactatgat	ARWIGEPQKEGYYYDYAMDV
AM947644.1	IGHV3-33*01	tgtgcgag		gtccagaaatgt	c	gggcattacggcg	ARSRNVGHYGAPDS
AM947644.1	IGHV3-33*01	tgtgcgag		gtccagaaatgt	c	gggcattacggcg	ARSRNVGHYGAPDS

AF471459.1	IGHV3-33*03	tgtgcgagaga		gtcacgggca		gtcgtggggg	ARESRAVVGGPFDY
AY515006.1	IGHV1-69*06	tgtgcgaga		ggggggcctgtgttgcagagtg		acgatttttggaatggttat	ARGGPVLQSDDFWNGYPPMDV
AY515006.1	IGHV1-69*06	tgtgcgaga		ggggggcctgtgttgcagagtg		acgatttttggaatggttat	ARGGPVLQSDDFWNGYPPMDV
AF471494.1	IGHV3-48*02	tgtgtgag		ggggaggtgggagacc		ggcgacggttgct	VRGRWETGDGCSFDV
AF471494.1	IGHV3-48*02	tgtgtgag		ggggaggtgggagacc		ggcgacggttgct	VRGRWETGDGCSFDV
AF471378.1	IGHV3-23*01	tgtgcgaaag		gggctggcagcgg		taggggtcggg	AKGAGSGRGRDYLDY
AF086903.1	IGHV3-30-3*01	tgtgcgaga		ggcgcgatgaaagac		tacgatttctggagtggtta	ARGAMKDYDFWSGYRHLGAFDI
AF471336.1	IGHV3-23*04	tgtacgaaag		gcgatactacgtc		gattcattactat	TKGDTTSIHYYYGMDV
AF471546.1	IGHV3-72*01	tgtactagag		gagccgcggcagcc		gcggcacttggt	TRGAAAAALGSLVYYYGMDV
L03175.1	IGHV3-30-3*01	tgtgcgag		gaacacccgagaaaac		atagaagcagatggtac	ARNTRENIEADGTAYYSYYMDV
L03165.1	IGHV3-15*06	tgtgccac		gaaatatccgcgctactcggata		tggtgactggtgttc	ATKYPRYSDMVTGVRNHFYMDV
L09098.1	IGHV3-15*06	tgtgccac		gaaatatccgcgctactcggata		tgatgactggtgttc	ATKYPRYSDMMTGVRNHFYMDV
AF471439.1	IGHV3-30*03	tgtgcgaaaga	tc	gcacgg		atagtgggaactac	AKDRTDSGNYDIDY
EU794437.1	IGHV5-51*03	tgtgcgaga		ctaggatatg		atgataccagtgattctt	ARLGYDDTSDSSASDFDY
EU794437.1	IGHV5-51*03	tgtgcgaga		ctaggatatg		atgataccagtgattctt	ARLGYDDTSDSSASDFDY
EU794398.1	IGHV1-69*01	tgt		ctgagaga		atattgtaatattgac	LREYCNIDNCRNFDH
AM947574.1	IGHV4-59*08	tg		cgtgagaagtcc		gacttttttt	VRSPTFFFNYFDF
AF471376.1	IGHV3-23*01	tgtgcgaaa		cggtcccgagaa		gggaggtac	AKRSREGRYGDS
AF471271.1	IGHV3-15*07	tgtaccaca		ccttggcgcgagatcc		gtatagcag	TTPWREIRIAGNAFDF
AF471385.1	IGHV3-23*01	tgtgcgaaa		cacacggaaa		tatggttcagggagt	AKHTEIWFRESSLDY
AF471385.1	IGHV3-23*01	tgtgcgaaa		cacacggaaa		tatggttcagggagt	AKHTEIWFRESSLDY
AF471291.1	IGHV3-23*01	tgtgtgaaa		cacacggaaa		tatggttcagggagt	VKHTEIWFRESSLDF
AF471291.1	IGHV3-23*01	tgtgtgaaa		cacacggaaa		tatggttcagggagt	VKHTEIWFRESSLDF
AF471232.1	IGHV3-9*01	tgtacaaaag		ccggatacggtc		gtatggcagtgg	TKAGYGRMAVGNWFDP
AF471232.1	IGHV3-9*01	tgtacaaaag		ccggatacggtc		gtatggcagtgg	TKAGYGRMAVGNWFDP
AM947617.1	IGHV1-46*01	tgtgc		ccgagagcctctaatg		atgatagtagtggtt	AREPLMMIVVVNWFDP
EU794412.1	IGHV3-7*01	tgtgcgagaga		atttagcagttatacgg		accacctggaata	AREFSSYTDHLEYYYDYYYMDV
AF471579.1	IGHV3-74*01	tgt		ataagaga		taaacaaggc	IRDKQGKDDY
AF471431.1	IGHV3-30*03	tgtgcgaaaga		aggagggagatttg		gtgcctcgtac	AKEGGRFGASYGAYFEY
AF471276.1	IGHV3-15*01	tgtaccacaga	t	aggagagcac	c	gtggatacagc	TTDRRAPWIQPQFDF
AF471167.1	IGHV3-7*01	tgtgcgaga		accgacggagg		gtctgcggg	ARTDGGSAGYFDF
EU794422.1	IGHV4-59*01	tgtgcgaga		acccagaccg		gcagcggctggtac	ARTQTGSGWYLED
EU794422.1	IGHV4-59*01	tgtgcgaga		acccagaccg		gcagcggctggtac	ARTQTGSGWYLED
AM947602.1	IGHV4-34*01	tgtgggaga		aatcccccgtcaaga		ctaagtgggga	GRNPPSRLSGDLRWRSGLPGRYFDL
EU794409.1	IGHV3-53*03	tgtgcgag		agagagggg		actatgacagcaatg	ARERDYDSNESGMDV
EU794409.1	IGHV3-53*03	tgtgcgag		agagagggg		actatgacagcaatg	ARERDYDSNESGMDV

GU980702.1	IGHV1-2*02	tgt		actagggaaaaaactgtgat		tacaattgggac	TRGKNCDYNWDFEH
GU980704.1	IGHV1-2*02	tgt		actagggaaaaa		attgtgattacaatt	TRGKNCDYNWDFEH
AF471456.1	IGHV3-33*03	tgtgc		aagagggaaccattg		gaccacagggact	ARGNHWTTGTAIDY
AF471456.1	IGHV3-33*03	tgtgc		aagagggaaccattg		gaccacagggact	ARGNHWTTGTAIDY
AF471267.1	IGHV3-15*01	tgtacca		aacacaa		ttcgggga	TKHNSGIDH
AF471225.1	IGHV3-9*01	tgcgcaaa		ggatatcaggagga		ggtataccagtggctggt	AKDIRRRYTSGWFPFDS
AF471333.1	IGHV3-23*04	tgtgcgagag		gagagggt		caatggctg	ARGEGSMAALYYFDY
AM947622.1	IGHV3-30-3*01	tgtgcgaga		gagagagac		gatattatagtggtggtagctgc	ARERDDIIVVVAALDY
AM947622.1	IGHV3-30-3*01	tgtgcgaga		gagagagac		gatattatagtggtggtagctgc	ARERDDIIVVVAALDY
AM947622.1	IGHV3-30-3*01	tgtgcgaga		gagagagac		gatattatagtggtggtagctgc	ARERDDIIVVVAALDY
AF471380.1	IGHV3-23*01	tgtgcgaa		gagagaca		ctagctgggga	AKRDTSWGFFDY
AF471380.1	IGHV3-23*01	tgtgcgaa		gagagaca		ctagctgggga	AKRDTSWGFFDY
M67504.1	IGHV5-51*03	tgtgcgaga		ctaggcaca		tattactatgatggtattccttat	ARLGTYYYDGIPYTYFDY
L38561.1	IGHV3-48*03	tgtgcgagaga	tc	ctaggag		gtggacacagctgtgg	ARDPRRWTQLWIPPDY
AF471544.1	IGHV3-72*01	a		ctagatccccgcg		ggggattg	RSPRGIGPHDF
EU794439.1	IGHV4-59*01	tgtgcg		cggattagtgttgttgtcctg		ccagctgttttccc	ARISVVVLPAVFPDI
AF471320.1	IGHV3-23*01	tgtgcgcaa		cacggacaacctt		atagagtggct	AQHGQPYRVAVDFSRF
AM947632.1	IGHV3-7*01	tgtgtgaga		cacggtgtg		ggcggtggctggttct	VRHGVGGGWFYFYYAMDV
AF471521.1	IGHV3-66*01	tgtgcgagaga		agaaattg		acaattactac	AREEIDNYYYGMDV

HCV

Accession #	V_H Gene	V_H	P	N1[a]	P	D_H	CDR3 (aa)[b]
AJ236548.1	IGHV1-69*01	tgtgcga		tgccctatccaaaac		attgcagtcgtggaagttgct	AMPYPKHCSRGSCWGWFDP
AF301517.1	IGHV3-48*02	tgtgcgagaga	t	tacggaggg		agcagtggttat	ARDYGGSSGYLNWFDP
AF301517.1	IGHV3-48*02	tgtgcgagaga	t	tacggaggg		agcagtggttat	ARDYGGSSGYLNWFDP
DQ179659.1	IGHV4-59*08	tgtg		ggcgactgtctaga		acggtgacta	GRLSRTVTMIDN
AF372844.1	IGHV3-15*01	tgtgccac		ggaatgtgccacggaatctctaatgg		atggcagcagctgg	ATECATESLMDGSSWTFDS
AY395753.1	IGHV1-69*06	tgtgcgaga		gaaggcatgagattacc		ccgaaacc	AREGMRLPRNPFDY
DQ073602.1	IGHV3-23*01	tgtgcga		gaacaggga		aggagtggctg	ARTGKEWLFPSDY
L26905.1	IGHV3-21*01	tgt		ctgagagggat		tatgttgactggtt	LRGIMLTGFWEGPGEHHYYGMDV
DQ179658.1	IGHV1-69*09	tgtgcga		catctcgcccggatac		aggattatttag	ATSRPDTGLFRKDAFDV
AF301519.1	IGHV3-7*03	tgtgcgagag		gagat		tattttgatgacg	ARGDYFDDDGPFIDVFNV

EBV

Accession #	V_H Gene	V_H	P	N1[a]	P	D_H	CDR3 (aa)[b]
M88316.1	IGHV4-39*01	tgtgcgcgac		cgctcacac		atgacgattttttgactgcttatta	ARPLTHDDFLTAYYPGGGYFDL
AB027447.1	IGHV4-39*01	tg		cacgagactaataaaag		attacaatggctggg	TRLIKDYNGWALFQQYYFMDV
AB027447.1	IGHV4-39*01	tg		cacgagactaataaaag		attacaatggctggg	TRLIKDYNGWALFQQYYFMDV
AB027441.1	IGHV3-30*04	tgt		ataagagatcaaact		tacggagtccac	IRDQTYGVHRFDS
AB027441.1	IGHV3-30*04	tgt		ataagagatcaaact		tacggagtccac	IRDQTYGVHRFDS
AF455924.1	IGHV3-23*04	tgtgcgaaaga	t	aggggcagg		atagcagcagct	AKDRGRIAAAHFDY
M88309.1	IGHV1-2*02	tgtgc		aattgaatacggggatactg		attgtggcggtgactgttactcc	AIEYGDTDCGGDCYSI
M88309.1	IGHV1-2*02	tgtgc		aattgaatacggggatactg		attgtggcggtgactgttactcc	AIEYGDTDCGGDCYSI
AB027445.1	IGHV3-20*01	tgtgcga		aattcgctagagcg		tggagtgg	AKFARAWSGPQFTDYYYYMDV

**Anti-Pneumococcal Ab**							
**Accession #**	**V_H Gene**	**V_H**	**P**	**N1[a]**	**P**	**D_H**	**CDR3 (aa)[b]**
DQ187521.1	IGHV3-11*01	tgtgcgaga		ccccgaaacataatcaagaggtggagcgg		ttactatgatagtag	ARPRNIIKRWSGYYDSRVPSEVDY
DQ187521.1	IGHV3-11*01	tgtgcgaga		ccccgaaacataatcaagaggtggagcgg		ttactatgatagtag	ARPRNIIKRWSGYYDSRVPSEVDY
DQ187521.1	IGHV3-11*01	tgtgcgaga		ccccgaaacataatcaagaggtggagcgg		ttactatgatagtag	ARPRNIIKRWSGYYDSRVPSEVDY
DQ187649.1	IGHV3-11*01	tgtgcgaga		acaaaaagaggga		attgtggtggtg	ARTKRGELWWWGNSFDY
DQ187649.1	IGHV3-11*01	tgtgcgaga		acaaaaagaggga		attgtggtggtg	ARTKRGELWWWGNSFDY
DQ187649.1	IGHV3-11*01	tgtgcgaga		acaaaaagagggga		attgtggtggtg	ARTKRGELWWWGNSFDY
DQ187649.1	IGHV3-11*01	tgtgcgaga		acaaaaagagggga		attgtggtggtg	ARTKRGELWWWGNSFDY
AF211206.1	IGHV3-15*01	tgtgcca		acgaaaac		ttttgg	ANENFWRLDN

The identified VH replacement products with the 5-mer footprints within the N1 regions from different diseased subcategories are listed under each diseased subgroups.

[a] The identified V_H replacement "footprints" are highlighted in *red* in the N1 regions.

[b] The amino acids encoded by the identified V_H replacement "footprints" are highlighted in *red* in the amino acid sequences of the CDR3 regions.

[c] One sequence with different potential 5-mer of V_H replacement footprints is included in a box.
